# Type IV Coupling Proteins as Potential Targets to Control the Dissemination of Antibiotic Resistance

**DOI:** 10.3389/fmolb.2020.00201

**Published:** 2020-08-12

**Authors:** Itxaso Álvarez-Rodríguez, Lide Arana, Begoña Ugarte-Uribe, Elena Gómez-Rubio, Sonsoles Martín-Santamaría, Carlos Garbisu, Itziar Alkorta

**Affiliations:** ^1^Department of Biochemistry and Molecular Biology, University of the Basque Country, Leioa, Spain; ^2^Department of Structural and Chemical Biology, Centro de Investigaciones Biológicas Margarita Salas, CIB-CSIC, Madrid, Spain; ^3^Department of Conservation of Natural Resources, Soil Microbial Ecology Group, NEIKER - Basque Institute for Agricultural Research and Development, Basque Research and Technology Alliance (BRTA), Parque Cient fico y Tecnológico de Bizkaia, Derio, Spain

**Keywords:** antibiotic resistance, bacterial conjugation, coupling proteins, drug discovery, novel conjugation inhibitors

## Abstract

The increase of infections caused by multidrug-resistant bacteria, together with the loss of effectiveness of currently available antibiotics, represents one of the most serious threats to public health worldwide. The loss of human lives and the economic costs associated to the problem of the dissemination of antibiotic resistance require immediate action. Bacteria, known by their great genetic plasticity, are capable not only of mutating their genes to adapt to disturbances and environmental changes but also of acquiring new genes that allow them to survive in hostile environments, such as in the presence of antibiotics. One of the major mechanisms responsible for the horizontal acquisition of new genes (*e.g*., antibiotic resistance genes) is bacterial conjugation, a process mediated by mobile genetic elements such as conjugative plasmids and integrative conjugative elements. Conjugative plasmids harboring antibiotic resistance genes can be transferred from a donor to a recipient bacterium in a process that requires physical contact. After conjugation, the recipient bacterium not only harbors the antibiotic resistance genes but it can also transfer the acquired plasmid to other bacteria, thus contributing to the spread of antibiotic resistance. Conjugative plasmids have genes that encode all the proteins necessary for the conjugation to take place, such as the type IV coupling proteins (T4CPs) present in all conjugative plasmids. Type VI coupling proteins constitute a heterogeneous family of hexameric ATPases that use energy from the ATP hydrolysis for plasmid transfer. Taking into account their essential role in bacterial conjugation, T4CPs are attractive targets for the inhibition of bacterial conjugation and, concomitantly, the limitation of antibiotic resistance dissemination. This review aims to compile present knowledge on T4CPs as a starting point for delving into their molecular structure and functioning in future studies. Likewise, the scientific literature on bacterial conjugation inhibitors has been reviewed here, in an attempt to elucidate the possibility of designing T4CP-inhibitors as a potential solution to the dissemination of multidrug-resistant bacteria.

## Introduction

Since their discovery, antibiotics have undoubtedly been one of the biggest, if not the biggest, medical revolutions. Regrettably, the abuse and misuse of antibiotics (mainly, for medicinal and veterinary purposes) has led to the emergence and dissemination of antibiotic resistant bacteria (ARB). The dissemination of antibiotic resistance is mainly driven by the horizontal transfer of antibiotic resistance genes (ARGs) using a variety of mobile genetic elements (MGEs), such as conjugative plasmids and integrative conjugative elements (ICEs) (Chan, 2015). In particular, biofilms represent a hot spot for horizontal gene transfer (HGT) by bacterial conjugation (Águila-Arcos et al., 2017).

Antibiotic resistance is responsible for the death of more than 33,000 people per year only in Europe, as well as a medical expenditure of around 1.5 billion euros (Cassini et al., 2019). It has been estimated that, by the year 2050, 10 million human lives might be at risk if no solution is found (Cassini et al., 2019). About 70% of hospital diseases are currently caused by multi-resistant bacteria (bacteria resistant to several antibiotics), further complicating their treatment. Not surprisingly, antibiotic resistance is at present one of the major threats to modern medicine and public health (World Health Organization, 2015, 2019a). In order to both inform and sensitize governments and the society in general, the World Health Organization presented a list of the most critical antibiotic-resistant pathogen species (Tacconelli et al., 2018). Although inevitably much more attention has been paid to the magnitude of this problem in hospital settings, the role of the environment on the emergence and dissemination of antibiotic resistance has been relatively recently recognized (Garbisu et al., 2018; Urra et al., 2019a,b,c).

Alas, the magnitude of this alarming problem has increased during the last years and decades, among other factors, due to the scarce presence of innovative proposals and treatments to fight ARB, as well as insufficient resources to carry out the required research to solve, or at least mitigate, the current antibiotic resistance crisis (Högberg et al., 2010; World Health Organization, 2019b).

At this point, it is essential to devise and develop new effective strategies in the fight against the emergence and dissemination of multi-resistant bacteria. Bacterial conjugation is one of the main mechanisms by which bacteria can acquire ARGs. Since type IV coupling proteins (T4CPs) are essential for the conjugation to take place, *a priori* they are attractive therapeutic targets for the inhibition of bacterial conjugation and, concomitantly, the limitation of antibiotic resistance dissemination. Therefore, in this review, in addition to presenting the existing knowledge on T4CPs, the latest advances in the search for inhibitors of bacterial conjugation will be described and discussed in an attempt to lay the foundations to facilitate the much needed work in the search for molecules that can efficiently inhibit T4CPs, thus opening the door to novel approaches and methodologies to control the spread of antibiotic resistance among bacteria.

## Horizontal DNA Transfer

Bacteria can modify their genetic material through three main mechanisms: (i) mutations that alter the DNA sequence; (ii) genetic rearrangements; and (iii) acquisition of new genetic material via HGT. Nowadays, it is a well-known fact that a high proportion of the bacterial genome corresponds to horizontally acquired genetic material. This fact highlights the key role of HGT for bacterial genome plasticity (Thomas and Nielsen, 2005; Johnsborg et al., 2007). Actually, bacteria can horizontally acquire genetic material through three different mechanisms: transformation, transduction, and conjugation (the latter is the most widespread mechanism of HGT) (Bello-López et al., 2019). A fourth, still scarcely understood, mechanism of HGT is mediated by bacterial membrane vesicles (Johnson and Grossman, 2015).

### Mobile Genetic Elements

The HGT processes of transduction and conjugation are mediated by MGEs. Mobile genetic elements have genes that encode the proteins necessary to move genetic material between different bacteria (intercellular transfer) or within the same bacteria among different DNA molecules (intracellular transfer) (Frost et al., 2005). Due to the complexity of the interactions among them, it is not easy to describe and unambiguously classify the variety of currently known MGEs. In any case, in general terms, the following groups can be differentiated: (i) bacteriophages; (ii) conjugative and mobilizable plasmids; (iii) genomic islands; (iv) transposable elements; and (v) integrons.

**Bacteriophages** (i.e., viruses that infect bacteria) can pack DNA segments of the host bacterium and transfer them to a new host bacterium, where they can be incorporated into its chromosome by recombination (Frost et al., 2005).

**Conjugative and mobilizable plasmids** are stable, self-replicating DNA molecules that carry genes not required for essential cellular functions but potentially useful in harsh conditions (Frost et al., 2005). Both types of plasmids can transfer genes between bacteria by means of using a type IV secretion system (T4SS) as conjugative machinery. But while conjugative plasmids code for all the proteins needed for self-transfer, mobilizable plasmids instead need a co-resident conjugative plasmid for them to be transferred by conjugation (Smillie et al., 2010).

**Genomic islands** are clusters of genes integrated into the bacterial chromosome through which bacteria can acquire advantageous functions such as, for instance, the ability to become infectious or resistant to antibiotics. Genomic islands are passively propagated during chromosomal replication, segregation and cell division (Bellanger et al., 2014). Two types of genomic islands can be distinguished: (i) ICEs, also known as conjugative transposons, contain a T4SS and, in consequence, they have the capacity to be transferred to other bacteria (Johnson and Grossman, 2015); and (ii) integrative mobilizable elements (IMEs) which encode their own excision, recircularization and integration sequences but lack some of the genes necessary for conjugative transfer and, hence, they need a co-resident ICE or conjugative plasmid for them to be transferred by conjugation (Bellanger et al., 2014).

**Transposable elements** are DNA sequences capable of moving themselves into different sites of the bacterial genome by (i) excision from the original site and insertion into a new site; or (ii) the generation of a new copy which will then be moved to a new site. This group of transposable elements includes (i) transposons (Tn) that, in addition to the genes specifically needed for transposition, encode other genes; and (ii) insertion sequences (IS) which only contain the genes required for transposition (Siguier et al., 2014).

**Integrons** can capture gene cassettes using site-specific recombination mediated by an integron-encoded integrase. Furthermore, they contain a promoter necessary for efficient transcription and expression of the gene cassettes. Relevantly, integrons can be embedded in different MGEs which they use for intra- and intercellular mobilization (Gillings et al., 2008).

For many years, it has been thought that conjugative plasmids were the main cause of HGT. Nevertheless, recent research has proven the relevance of ICEs (integrative conjugative elements) and IMEs (integrative and mobilizable elements) for HGT (Guédon et al., 2017). In any case, since conjugative plasmids have genes that encode all the elements required for conjugation, they have been studied thoroughly and accurately.

### Type IV Secretion Systems

Type IV secretion systems (T4SS) are macromolecular assemblies that can transport DNA and/or proteins (Christie, 2016). This broad superfamily, in terms of function and structure, of macromolecular complexes can be found in both Gram-negative and Gram-positive bacteria (Christie et al., 2017). Different classifications, based on different criteria, have been proposed for T4SSs (Table 1). Although they do not cover all the cases, due to the great variability observed within this superfamily, two main classifications are regularly used. The first classification is based on the T4SS function and differentiates two types (de Paz et al., 2005): T4SS that take part in conjugation (cT4SS) and T4SS that take part in pathogenic processes (pT4SS). In any case, some T4SS, such as the one present in *Agrobacterium tumefaciens*, show a dual nature (Li and Christie, 2018). The second classification is based on the protein components (Christie et al., 2017; Grohmann et al., 2018). In this case, T4SS are similarly divided into two groups: type IV A (T4ASS) and type IV B (T4BSS), whose paradigmatic systems are the VirB/VirD system of *A. tumefaciens* and the Dot/Icm system of *Legionella pneumophila*, respectively. T4ASS are composed of approximately 12 proteins related to the VirB-D proteins of *A. tumefaciens* (Christie et al., 2005). Although T4ASS proteins are termed VirB1-11, there is a tendency to use TivB1-11 nomenclature, instead of VirB, to avoid confusion (Thomas et al., 2017). T4ASS are associated with the dissemination of ARGs. On the other hand, T4BSS are composed of 25 proteins, of which only a few are homologous to the VirB system (Voth et al., 2012).

**TABLE 1:** Classifications of T4SSs.

Criterion	Sub-families	Reference
Pilus type	F-like, P-type, I-type	Lawley et al., 2003
Relaxase phylogeny	MOB(F), MOB(H), MOB(Q), MOB(C), MOB(P), MOB(V)	Garcillán-Barcia et al., 2009
Substrate	Relaxase and ssDNA (conjugative systems), effector translocator systems, DNA release and uptake systems	Alvarez-Martinez and Christie, 2009
VirB4 phylogeny	MPF_*I*_, MPF_*C*_, MPF_*G*_, MPF_*T*_, MPF_*F*_, MPF_*B*_, MPF_*FATA*_, MPF_*FA*_	Guglielmini et al., 2013
Function	cT4SS, pT4SS	de Paz et al., 2005
Protein components	T4ASS, T4BSS	Christie et al., 2017; Grohmann et al., 2018

**FIGURE 1:**
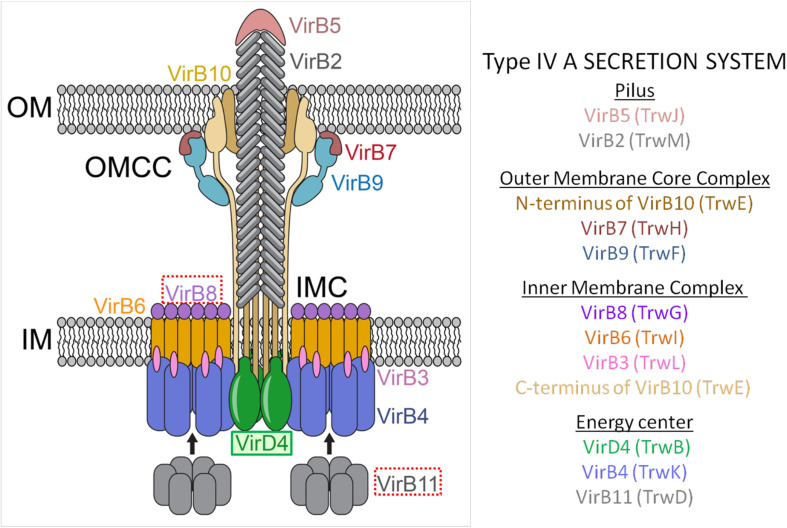
Molecular architecture of the T4ASS showing the location of the T4CP. Different components of the pilus, OMCC, IMC, and the ATPase energy center are shown. To show equivalence between VirB/VirD4 and R388 systems, proteins corresponding to R388 are shown in brackets. Arrows represent dynamic interactions of VirB11 protein through the conjugative process. VirD4 is highlighted in green. Red boxes highlight the proteins selected as targets for drug discovery (VirB8 and VirB11). OMCC, outer membrane core complex; IMC, inner membrane complex. The schematic representation is based on the structure described by Redzej et al. (2017). The image has been adapted from Waksman (2019).

Gram-negative T4ASS evolved as supramolecular protein structures formed by three different functional units or modules (Figure 1). The first module, formed by three ATPases [VirD4 (T4CP), VirB4, VirB11], is an energy center located in the cytoplasmic side at the entrance of the secretion channel (Ripoll-Rozada et al., 2013). These proteins interact with the second module, a larger infrastructure called the inner membrane complex (IMC) (Low et al., 2014), formed by VirB3, VirB6, VirB8, and the N-terminus of VirB10. The IMC, responsible for substrate translocation through the inner membrane (IM), is connected to the outer membrane core complex (OMCC), i.e., the third module. The OMCC, formed by VirB7, VirB9, and the C-terminus of VirB10, is responsible for substrate translocation through the periplasm and the outer membrane (OM) (Gordon et al., 2017). Finally, the conjugative pilus, a structure essential for the direct contact between bacterial cells (Lawley et al., 2003), is composed of VirB2 and VirB5 proteins. Recent structural studies on the F-plasmid have provided new insights into the different types of channel structures and conjugative pili that can be present in donor bacteria (Hu et al., 2019a).

In Gram-positive bacteria, the OMCC is not needed and, consequently, T4ASS are minimized. Actually, in Gram-positive bacteria, the OMCC is composed of only six VirB/VirD4 proteins and seems to lack a pilus-like structure. Instead, Gram-positive bacteria use adhesins for cell-to-cell contact (Grohmann et al., 2017). Recently, it has been reported (González-Rivera et al., 2019) that the Gram-negative pKM101 plasmid can also use adhesin-like structures instead of a conjugative pilus, a mechanism until now observed only in Gram-positive bacteria.

### Conjugative and Mobilizable Plasmids

Plasmids are autonomously replicating circular DNA molecules located in the bacterial cytoplasm. It has been estimated (Smillie et al., 2010) that a quarter of the plasmids are conjugative plasmids, another quarter corresponds to mobilizable plasmids and, finally, half of all plasmids are non-mobilizable plasmids. Together, conjugative and mobilizable plasmids constitute the so-called transmissible plasmids. In general, plasmids provide advantageous properties and capacities for bacterial survival such as, for instance, resistance to bactericidal and bacteriostatic antibiotics. Plasmids are classified according to Incompatibility Groups (Inc) so that two plasmids of the same group cannot coexist at the same time within the same bacterial cell. Some Inc groups (e.g., IncN, IncP, and IncW) can be found in many different bacterial species; others (e.g., IncF and IncI), in contrast, can only be stably kept in specific bacterial species (Kittell and Helinski, 1993). Conjugative and mobilizable plasmids contain all or some of the genes that take part in the conjugation process, respectively.

**FIGURE 2:**
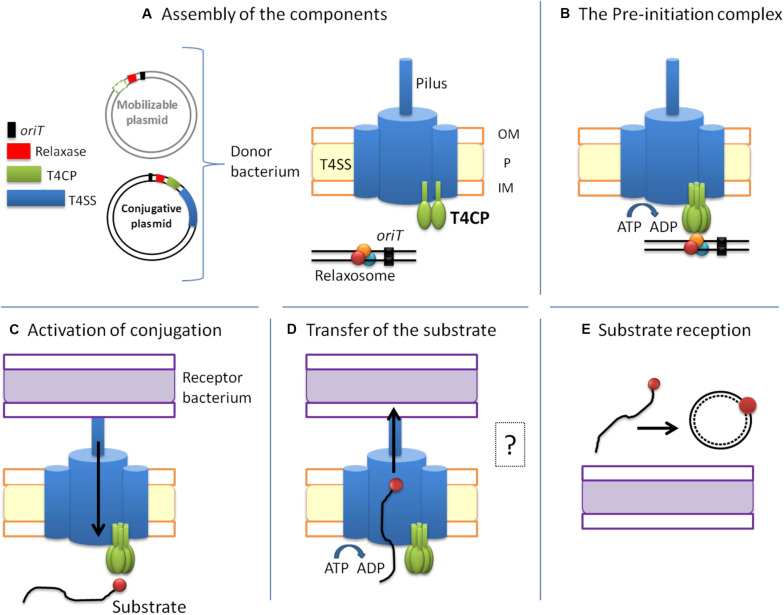
The conjugative process. Conjugative plasmids encode all the elements necessary for conjugation, while mobilizable plasmids lack a cognate T4SS and most of them also lack the T4CP. The conjugative process can be divided into five main steps: **(A)** Assembly of the components. **(B)** Formation of the pre-initiation complex. The T4CP performs the reception of the relaxosome to the secretion channel. This interaction triggers the hexamerization of the T4CP, the ATP hydrolysis by T4CP and VirB11, and finally the activation of the secretion channel. Both conjugative and mobilizable plasmids employ the T4CP of a conjugative plasmid, except for the few mobilizable plasmids that encode their cognate T4CP. **(C)** Activation of the conjugative process and processing of the substrate. When the pilus recognizes a receptor bacterium, a signal is transmitted to the relaxosome and DNA is processed. Each relaxase accomplishes the processing of its cognate plasmid through the specific recognition of the *oriT* sequence. **(D)** Transfer of the substrate through the T4SS. This step is always accomplished *via* the T4SS encoded in the conjugative plasmid. The question mark indicates that many questions remain open. **(E)** Substrate reception and end of conjugation. Once the substrate enters the recipient bacterium, the ssDNA is recircularized and replicated. Color code: *oriT*, black; relaxase, red; T4CP, green; T4SS and pilus, blue; donor bacterium, orange; receptor bacterium, purple.

Conjugation-related genes are divided into two modules: MOB (mobility) and MPF (mating pair formation) genes (Smillie et al., 2010; Figure 2). Conjugative plasmids have both modules and, therefore, harbor genes that encode all the proteins needed for HGT. The MOB module, formerly called DNA Transfer and Replication (Bhatty et al., 2013), consists of three sequences necessary for substrate processing. First, the *oriT* (origin of transfer) sequence, which marks the starting point for DNA processing. Second, the sequence that encodes the relaxase which specifically recognizes the *oriT* sequence of its plasmid. Relaxase is the only conserved protein in all transmissible plasmids. The third protein encoded in the MOB module is T4CP, which recognizes the relaxosome in the cytosol and connects it with the secretion channel located in the membrane. Likewise, accessory proteins that take part in the processing of the relaxosome can also be found in the MOB module. On the other hand, the MPF module encodes the proteins that build the T4SS (VirB1-11 in the case of T4ASS).

Mobilizable plasmids need a co-resident conjugative plasmid for them to be transferred because they lack the MPF module and contain only some of the sequences described above for the MOB module. In particular, all mobilizable plasmids contain the *oriT* and the relaxase which are specific to each plasmid. Some mobilizable plasmids also contain a T4CP. Mobilizable plasmids that encode their own T4CP form the MOBC1 plasmid subfamily, with plasmid CloDF13 as the prototype of this subfamily (Garcillán-Barcia et al., 2009).

### Conjugation

Bacterial conjugation was initially described as a mechanism of sexual reproduction among bacteria (Lederberg and Tatum, 1946). This mechanism of HGT originally emerged in ancient proteobacteria and then expanded to all prokaryotes. Thanks to the new horizontally acquired genes, the recipient bacterium can acquire novel traits that allow it to survive in challenging environments such as, for instance, in the presence of antibiotics.

Bacterial conjugation requires direct physical contact between the two bacterial cells so that the ssDNA-protein complex can be transferred from the donor to the recipient bacterium. This process is usually divided into three steps: (i) the formation of the relaxosome; (ii) the reception of the substrate to the secretion channel; and (iii) the translocation of the substrate from the donor to the recipient bacterial cell (Alvarez-Martinez and Christie, 2009). However, recent studies have suggested additional steps: first, to provide the required cell-to-cell contact that initiates the transfer of the substrate, before the formation of the relaxosome, the secretion channel has to be established and the pilus formed. Also, during substrate translocation, the machinery has to switch from a protein transfer mode to a ssDNA transfer mode (Waksman, 2019). All these steps reflect the highly complex nature of the conjugative process, with many actions happening simultaneously.

A high amount of energy is required for conjugation to occur, which explains the presence of different ATPases within the conjugative machinery. In order to perform the transfer of the substrate with the proper energy expenditure, the conjugative process is highly regulated. Although there are certainly still many unclear and unresolved aspects, in this review we have divided the conjugative process into five main steps (Figure 2) which apply to conjugation in Gram-negative bacteria (the conjugation process is slightly different in Gram-positive bacteria) (Grohmann et al., 2018):

**A. Assembly of the T4SS channel, the conjugative pilus and the relaxosome.** Many unknown signals trigger the formation of the T4SS secretion channel. In the canonical T4SS of *A. tumefaciens*, *virB* gene expression is activated through the sensing of an external factor from the milieu, which could also be the case for other systems (Li and Christie, 2018). For the establishment of the physical contact between donor and recipient cells, the pilus (a polymer of VirB2 subunits) is then assembled. Additionally, the relaxosome (composed of the relaxase, DNA and accessory proteins) has to be formed in the cytoplasm (Figure 2A).

**B. Formation of the pre-initiation complex.** Once the pilus, the T4SS and the relaxome are formed, they all interact to form a pre-initiation complex which stays latent until conjugation begins (Waksman, 2019). The relaxosome is recruited by the T4CP through a variety of interactions. Although relaxases are known to possess translocation signals in their sequence for T4CP recognition (Christie, 2004), they are not the main responsible elements for the abovementioned interaction. Actually, the T4CP establishes the strongest interactions with the accessory proteins (Llosa and Alkorta, 2017). The structural basis of these interactions has not been resolved to date. Therefore, much further research is needed to better understand this mechanism (Li et al., 2019). It has been postulated (Waksman, 2019) that, as a consequence of those interactions, the T4CP either hexamerizes or creates heterohexamers with VirB4, which is attached to the secretion channel. The binding of DNA to the T4CP and the subsequent interaction with the T4SS appear to trigger the hydrolysis of ATP by the T4CP and the VirB11-like protein. These processes induce a conformational change in VirB10, thus activating the secretion channel (Cascales and Christie, 2004a; Cascales et al., 2013; Figure 2B).

**C. Activation of the conjugative process.** Although intracellular signals caused by DNA-binding and ATP-hydrolysis are necessary for substrate transfer, an extracellular signal is also required. This extracellular signal is generated when the pilus recognizes a recipient cell (Zechner et al., 2012). Once the physical contact is established, the pilus retracts (F-type) or falls (P-type), forming the junction between the two bacterial cells (Lawley et al., 2003; Schröder and Lanka, 2005). Moreover, the recognition signal is transduced through the T4SS to the cytoplasm, thanks to a series of interactions between VirB10-like proteins and T4CP (Llosa and Alkorta, 2017). As a consequence, the relaxase nicks one of the DNA strands and gets covalently attached to the 5′ end. Further, DNA replication takes place as a rolling circle replication, forming the DNA strand from the free 3′ end (Llosa et al., 2002; Figure 2C).

**D. Substrate transport through the T4SS.** The substrate is composed of the relaxase covalently bound to ssDNA. To transfer a complex of such different characteristics (i.e., protein and nucleic acid), the T4SS needs to undergo substantial changes during conjugation. Many questions remain unanswered on this matter, e.g., Which component is transferred first? How is the relaxase unfolded and transferred? According to the transfer DNA immunoprecipitation (TrIP) assay, the ssDNA is transferred from T4CP to VirB11 without energy cost. Then, the substrate is transferred to VirB6 and VirB8 proteins. At this moment, the ATPases need to be active to generate the required energy. Next, the substrate is transferred from VirB8 to VirB9 and, finally, to VirB2 in the pilus (Cascales and Christie, 2004b). However, the substrate pathway solved by the TrIP does not match the existing structural models. In consequence, this matter should be further explored according to new pieces of evidence (Waksman, 2019). Although the interactions between the substrate and the different proteins have already been defined and described, the translocation mechanism is not well established yet. Originally, one-step and two-step models were proposed. In the first model (one-step), the ssDNA substrate would be transferred directly to the T4SS whereas, in the second model (two-step), the ssDNA substrate would be transferred from the T4CP channel to the periplasm, and from there to the T4SS (Llosa et al., 2002). In any case, this second model has been discarded due to the high content of nucleases present in the periplasm. Instead, it has been proposed (Christie, 2016) that the T4CP is placed at the entrance of the T4SS, translocating the substrate directly to the T4SS. Three possible translocation pathways have been proposed for the ssDNA substrate: (i) translocation could occur, through the channel of the T4CP hexamer, to the T4SS; (ii) after its uptake by the T4CP, the substrate could pass through the VirB4 hexamer and from there it would be translocated to the T4SS; or (iii) after its interaction with the ATPases, the substrate could be translocated directly through a channel formed by IMC subunits (Christie et al., 2017). In any case, many open questions remain to be answered about the conjugative process (Figure 2D).

**E. Substrate reception and end of conjugation.** Although substrate transport through the T4SS has been relatively well studied, it is currently not clear how the substrate crosses the outer and inner membrane of the recipient cell. Once in the recipient cell, the relaxase circularizes the lineal ssDNA, which is then used as substrate for the synthesis of the complementary strand (to obtain the dsDNA plasmid), a process in which the relaxase itself may also take part (Draper et al., 2005; Figure 2E).

Thanks to the conjugative process, the recipient cell benefits from the characteristics encoded in the acquired plasmid. In particular, if the recipient cell has acquired a conjugative plasmid, it gains the ability to transfer it to other bacteria, thus becoming a donor bacterial cell.

## Coupling Proteins

Type IV coupling proteins are essential for most T4SSs. As a matter of fact, T4CPs are (i) key players in all the conjugative systems; and (ii) present in many pathogenic processes that translocate effector proteins. As indicated above, T4CPs are (i) part of the MOB module in transmissible plasmids; and (ii) normally encoded in juxtaposition with relaxases. The phylogeny of T4CPs shows strong similarity with the phylogeny of relaxases, suggesting that substrates might have evolved along with their receptors. Concerning phylogeny, T4CPs are good markers of conjugative systems because of their ubiquity and size (Smillie et al., 2010). The most studied T4CPs are those associated with T4ASS of Gram-negative bacteria (e.g., VirD4_*At*_, TrwB_*R*388_, and TraD_*F*_). Nonetheless, studies have been extended to T4CPs from Gram-positive bacteria (e.g., PcFC_*pCF*10_ and TcpA_*pCW*3_) (Parsons et al., 2007; Chen et al., 2008) and T4BSSs of Gram-negative bacteria, such as DotL_*Lp*_ (Sutherland et al., 2012; Meir et al., 2018).

The study of T4CPs has a double purpose. On the one hand, taking into account their essential role in conjugation, they have become interesting potential targets for the development of conjugation inhibitors, as it is the case of other T4SS components (Shaffer et al., 2016). In this manner, the spread of ARGs could be controlled (similarly, in the case of pT4SSs, pathogenic processes could be prevented). On the other hand, the specific substrate transport carried out by T4CPs offers a wide range of possibilities in the field of biotechnology, including the targeted transport of desired molecules through heterologous systems (Guzmán-Herrador et al., 2017).

### Classification

Type IV coupling proteins are a family of heterogeneous proteins that show lower sequence similarity than expected taking into account their phylogenetic proximity. The motif that defines the members of this family is the conserved Nucleotide-Binding Domain (NBD). Nonetheless, different domain architectures can be found among T4CPs. For instance, most T4CPs are membrane proteins that show a transmembrane domain (TMD) at the N-terminal end, as well as a cytoplasm-oriented all-alpha domain (AAD), but, in some cases, they can also present a variable domain in the C-terminal domain (CTD).

**FIGURE 3:**
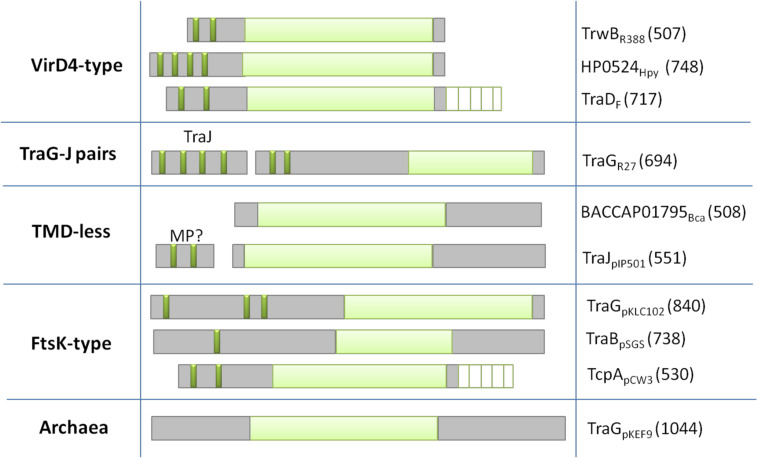
Classification of T4CPs according to their molecular architecture. Each line represents one of the five subfamilies. Examples of each family are shown in the second (molecular architecture) and third (protein name and sequence length) column. Dark green, transmembrane helices; light green, nucleotide-binding domain; white lined squares, C-terminal domain. MP?, membrane protein postulated to interact with the TMD-less T4CP to perform its *in vivo* activity.

Based on the architecture and nature of these domains, T4CPs were classified into six subfamilies (Alvarez-Martinez and Christie, 2009) that were later reduced to five (Llosa and Alkorta, 2017; Figure 3).

#### VirD4-Type

This is the first described and most studied subfamily of T4CPs (Christie, 2016). Its name is given by the T4CP of the *A. tumefaciens* T-plasmid (i.e., VirD4_*At*_). TrwB_*R*388_, the prototype of the T4CP family, is included within this VirD4-type subfamily (Christie, 2004). The VirD4-type subfamily includes membrane proteins of 500–750 residues which show, at least, two transmembrane helices at the N-terminal end. They have a low sequence identity among themselves (around 15–20%). However, it has been observed (Cabezón et al., 1994; Llosa et al., 2003) that some of these proteins can be interchanged to perform the conjugation of mobilizable plasmids *in vivo.*

#### TraG-J Pairs

These are membrane proteins with a similar structure to VirD4-type T4CPs, a length of around 700 residues, and shorter periplasmic domains. They present higher sequence similarity among themselves and, like VirD4-type T4CPs, they can be functionally interchanged. They need to interact with a membrane protein, i.e., TraJ, encoded next to the T4CP gene, in order to perform their function (Gunton et al., 2007). TraJ-like proteins have a length of approximately 200 residues and up to five transmembrane helices.

#### T4CP Lacking TMD

The members of this subfamily do not have a TMD (this TMD is essential for the function of most T4CPs). However, some of these proteins interact with small membrane proteins, forming complexes similar to VirD4-type T4CPs. Two of the most studied proteins within this subfamily are TraJ_*pIP*501_ (formerly called Orf10_*pIP*501_) and TraI_*pIP*501_ (formerly called Orf9_*pIP*501_) (Grohmann et al., 2016).

#### FtsK-Type T4CP

It has been reported (Gomis-Rüth and Coll, 2006) that the structure of T4CPs shows certain similarities with the structure of translocases from ssDNA SpoIIIE/FtsK. This similarity is increased in the case of FtsK-type T4CP. FtsK-type T4CPs have more than 750 residues and several TMDs separated from the NBD by large linker sequences. Although they do not show any AAD, they usually have an additional domain for ATP-binding and hydrolysis (Parsons et al., 2007).

#### Archaea T4CP

In the Archaea domain, some T4CPs have been identified by predicting sequences based on NBDs (Alvarez-Martinez and Christie, 2009).

### Structure

As mentioned before, almost all T4CPs are composed of a TMD and a cytosolic domain. The cytosolic domain contains an NBD, an AAD and, in some cases, a CTD. The prototype of these T4CPs is TrwB_*R*388_. The structure of its soluble mutant protein (i.e., TrwBΔN70) has been resolved by X-ray crystallography (Gomis-Rüth et al., 2001).

#### Domains

##### Transmembrane domain

For VirD4-type T4CPs, the TMD is essential for *in vivo* conjugation (Chen et al., 2008; Segura et al., 2013). This domain is involved in the interaction between the T4CP and VirB10 protein (Llosa et al., 2003; Segura et al., 2013), which is crucial for the activation of the T4SS. Besides, the TMD is critical for protein oligomerization as it contains key residues for such process (Redzej et al., 2017). The TMD is formed by α-helices and contains periplasmic loops essential for T4SS interactions and T4CP functions (Kumar and Das, 2002; Gunton et al., 2005).

The TMD of TrwB_*R*388_ is the most extensively studied. Actually, it is the only T4CP that has been purified in its native version. In those studies, it has been demonstrated that (i) a progressive elimination of TMD elements leads to a reduction in the frequency of DNA transfer; and (ii) the mutant protein lacking the TMD (TrwBΔN70) has no DNA transfer activity (Segura et al., 2013). Likewise, the effect of the TMD on the protein structure was analyzed by infrared spectroscopy (Hormaeche et al., 2004; Vecino et al., 2011), observing that TrwB_*R*388_ is more stable than TrwBΔN70 under denaturing conditions (i.e., low ionic force, presence of chaotropic agents, high temperature), thus suggesting that the TMD contributes to a more compact and ordered folding of the cytosolic domain. The TMD also has an effect on nucleotide-binding activity, even though this activity does not happen in this TMD. The wild-type protein presents a lower binding affinity to all nucleotides but a higher specificity to purine nucleotide triphosphates (Hormaeche et al., 2006). It seems that the presence of the TMD reduces the accessibility of the NBD, thus becoming more specific for ATP. Still, TrwBΔN70 has DNA-dependent ATPase activity, which is enhanced in the presence of TrwA_*R*388_ (this activity has not been detected in the native protein) (Tato et al., 2007). All these results suggest that the TMD could have a regulatory function in the cytosolic domain. Finally, the native protein, as well as the TMD by itself, is located in the cell poles, whereas the soluble mutant protein, in the absence of other conjugative proteins, is found in the cytoplasm. By contrast, in the presence of T4SS proteins, TrwBΔN70 is evenly distributed along the cell periphery, suggesting that the TMD leads the polar location but interactions between the cytosolic domain and T4SS proteins contribute to the membrane location of TrwB_*R*388_ (Segura et al., 2014).

Despite its crucial role, the TMD shows the most heterogeneous sequence among T4CPs. Although critical for the activity of VirD4-type T4CPs, T4CPs that lack a TMD are capable of accomplishing the transfer of their cognate plasmids. Moreover, the TMD-lacking mutant protein of FtsK-type TcpA_*pcW*3_ T4CP, i.e., TcpA_Δ46–104_, can certainly perform conjugation, although at a lower frequency than the wild-type protein (Parsons et al., 2007). Comparative studies between TrwB_*R*388_ and TrwBΔN70 have shed light on the role of the TMD, which goes beyond being a simple anchor of TrwB_*R*388_ to the membrane, something that may be also true for other T4CPs. The study of membrane proteins is a challenging task. In consequence, most studies on T4CPs have been performed using soluble mutant versions. Accordingly, more comparative studies using wild-type and soluble-mutant proteins from different members of the T4CP family are highly required.

##### Cytosolic domain

The cytosolic domain can be divided into two subdomains: NBD and AAD. Occasionally, a third domain, CTD, is also present.

###### Nucleotide-binding domain

The NBD, the most conserved domain, is shared by all T4CPs. This domain has two motifs. The first motif, i.e., P-loop or Walker A, binds β and γ phosphates of nucleotides (Walker et al., 1982). This P-loop motif, usually represented as G-x(4)-GK-(TS), where x can be any amino acid, is rich in glycine and has a conserved lysine followed by a serine or a threonine. The P-loop motif is present in many ATP- or GTP-binding proteins such as, for instance, the α and β subunits of ATP synthases, myosin, adenylate cyclase, and the ABC protein family (Geourjon et al., 2001). The second motif is called Walker B and its sequence is represented as (RK)xxxGxxx-LhhhDE, where x refers to any amino acid and h is a hydrophobic amino acid (Walker et al., 1982; Hanson and Whiteheart, 2005). Topologically, the NBD is located near the membrane while, structurally, it is formed by a twisted β-sheet surrounded by α-helices (Gomis-Rüth and Coll, 2006). This conformation resembles that of the β-domain of FtsK protein, a membrane protein motor that drives DNA across the membrane during cell division (Aussel et al., 2002). The NBD is essential for the *in vivo* activity of T4CPs. In fact, Walker A mutants cannot accomplish conjugation (Moncalián et al., 1999; Kumar and Das, 2002; Gunton et al., 2005), possibly due to the lack of ATPase activity (Tato et al., 2005). This lack of ATPase activity does not seem to affect other characteristics of the mutant proteins such as cell location, nucleotide-binding affinity, and interaction with the substrate (Moncalián et al., 1999; Chen et al., 2008). These findings suggest that T4CP function could take place after nucleotide- and substrate-binding events.

##### All-alpha domain

The AAD is oriented toward the cytoplasm and presents a variable size, sequence and folding pattern. This domain is also present in many proteins from the RecA family (Gomis-Rüth et al., 2001). Furthermore, the AAD domain of TrwB_*R*388_ presents structural similarities to the N-terminal domain of the XerD recombinase, a protein that interacts with the previously named FtsK resolving the DNA knots formed after replication. As the XerD’s N-terminal domain is located over the NBD, it blocks DNA-binding and, therefore, a conformational rearrangement is needed to interact with the substrate. A similar situation could happen in T4CPs, where the AAD would block the NBD and, after a conformational change, could take part in DNA-binding (Gomis-Ruth et al., 2005). Besides, the six α-helices present in AAD of TrwB_*R*388_ show similar topological characteristics to the DNA-binding domain of TraM_*F*_, a component of the relaxosome of the F-plasmid that interacts with T4CPs. No homologous of TraM_*F*_ has been found in the R388 plasmid, suggesting that TrwB_*R*388_ has incorporated the structure of this interacting domain for DNA-binding. Whitaker et al. (2015) reported two essential roles of the AAD in bacterial conjugation: (i) it activates the secretion channel; and (ii) it specifically recognizes its cognate relaxosome and accessory proteins. Mobile genetic elements that lack a cognate T4CP must then overcome this specificity in order to be transferred through a heterologous T4SS.

##### C-terminal domain

Some T4CPs, such as VirD4-type (e.g., VirD4_*At*_), FtsK-type (e.g., TcpA_*pCW*3_), and Archaea-type (e.g., TraG_*pKEF*9_), acquired a new CTD (Alvarez-Martinez and Christie, 2009). CTMs present high heterogeneity in terms of sequence and size, but all of them have glutamate- and aspartate-enriched regions.

The CTDs of different conjugative systems can present different functions. Many DNA-binding proteins show acidic C-terminal sequences that take part in the DNA-binding process (Lee and Thomas, 2000; Wang et al., 2007; Harrison et al., 2016; Basu et al., 2020), which appears to be their main function in T4CPs. For example, in the case of TraD_*F*_ T4CP, the interaction of its CTD with one of the components of the relaxosome, TraM_*F*_, is essential for recognition and plasmid transfer (Lu and Frost, 2005; Lu et al., 2008). In a similar manner, the CTD domain of TcpA_*pCW*3_ triggers a threefold enhancement of conjugation frequency via its interaction with other T4SS proteins (Steen et al., 2009).

Some T4CPs are related with pathogenic systems that contain a CTD. On the one hand, Whitaker et al. (2016) reported that the relevance of the CTM in VirD4_*At*_ depends on the substrate. These authors concluded that the CTD of VirD4-type T4CPs (related to pT4SS) evolved to (i) widen the effector repertoire; and (ii) improve the space-time regulation of the effector presentation. On the other hand, it has been described (Sutherland et al., 2012; Meir et al., 2018) that the acidic tail of protein DotL_*Lp*_ from *L. pneumophila* is involved in the reception of the effector proteins from the IcmSW system.

#### Structure of TrwB_*R*388_

Up to date, only two crystal structures related to T4CPs have been achieved: (i) the crystal structure of the TMD-less variant of TrwB_*R*388_, TrwBΔN70 (Gomis-Rüth et al., 2001); and (ii) the C-terminal extension of DotL_*Lp*_, as part of the Dot/Icm holocomplex from the T4BSS of *L. pneumophila* (Kwak et al., 2017). Since TrwB_*R*388_ is the most studied T4CP, it is considered the archetypal of this protein family. The obtained structure is similar to TrwK_*R*388_ structure (a homolog of VirB4 in the R388 system), which underlines the structural homology between VirD4 and VirB4-like proteins, despite their poor sequence similarity, and reinforces their phylogenetic relationship (Walldén et al., 2012; Guglielmini et al., 2013). In fact, the X-ray crystallographic structure of TrwBΔN70 (PDB ID 1E9R) was used to construct, by homology modeling, a 3D model for VirB4 (Middleton et al., 2005). These authors suggested that VirB4 may act as a docking and pumping agent at the entrance of the T4SS channel, in concert with other T4SS components, to transport substrates through the T4SS. Subsequent structural and mechanistic studies have demonstrated that T4CPs are located in between the VirB4 ATPases (Redzej et al., 2017).

TrwBΔN70 has been crystallized without ligands or in the presence of nucleotides and sulfate ions (Gomis-Rüth et al., 2001, 2002b). Its structure shows similarities with other protein families, such as AAA+ ATPases, RNA and DNA helicases, and proteins from the FtsK/SpoIIIE family. TrwBΔN70 has a homohexameric globular structure (110 Å in diameter and 90 Å in height) with an internal channel (ICH) that goes across the hexamer (a 22 Å wide in the membrane side and 7 Å wide in the cytosolic side). This narrowing from 22 to 7 Å in the cytosolic side suggests the existence of conformational changes to enable the substrate to enter into the transport system (Schröder and Lanka, 2005). By means of electrostatic potential calculations, it has been described (Larrea et al., 2017) that there are three electrostatic regions inside the channel: (i) a short hydrophobic region in the cytosolic side; (ii) a highly electronegative segment in the middle part of the channel; and (iii) an electropositive part in the second half of the channel. The functional relevance of the different amino acids has been revealed by the analysis of mutants, concluding that amino acids at both ends of the ICH regulate the opening of the channel and that amino acids in the middle part of the channel define the translocation pathway of the substrate (Larrea et al., 2017).

**FIGURE 4:**
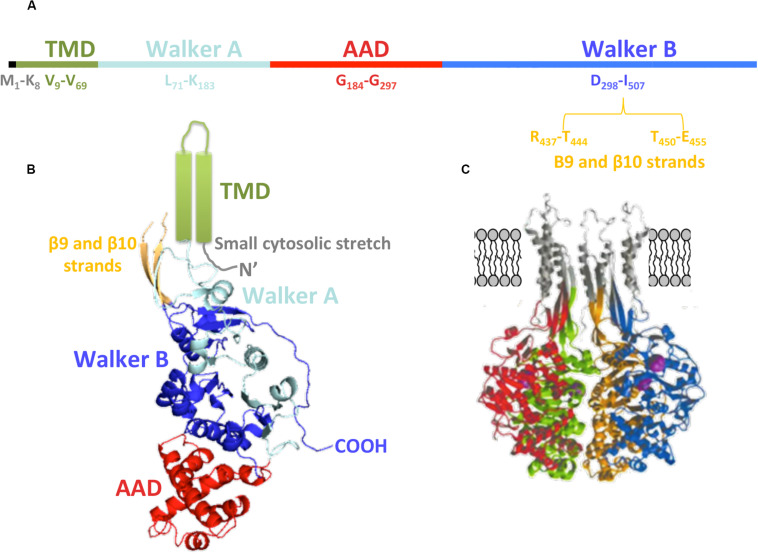
TrwB_*R*388_ structure. **(A)** Primary structure. The location of the domains along the sequence is shown. **(B)** Diagram of TrwBΔN70 monomer. The different domains are colored as in panel **(A)**. The TMD (green), not solved in the crystal structure, is represented as two cylinders. Regarding β9 and β10 strands (orange), only their structurally solved parts are shown (PDB: 19E6). **(C)** Representation of four monomers (each of them in a different color) with *in silico* modeled TMD, β9, and β10 strands (gray). Panel **(C)** adapted from Gomis-Rüth et al. (2002a).

Regarding the primary structure of TrwB_*R*388_ (Figure 4A), the first amino acids (M_1_ to K_8_) are related to a small cytosolic domain, followed by the TMD (V_9_ to V_69_). According to its sequence, this region is formed by two α-helices linked by a loop that is proposed to be located in the periplasmic region, perhaps interacting with the T4SS and with periplasmic regions of other subunits of the hexamer. Walker A and Walker B domains are located from L_71_ to K_183_ and from D_298_ to I_507_ amino acids, respectively. The AAD is located between both Walker domains (G_184_-G_297_) (Figure 4A). The region between R_437_-T_444_ and T_450_-E_455_ amino acids, related to the membrane-proximal edge of β9 and β10 strands, has not been solved in the soluble mutant protein structure, probably due to its interaction with the excised TMD (Figure 4B). Modeling of this region rendered β-sheets that, together with the α-helices of the TMD, could create the right channel for substrate transport through the membrane (Figure 4C).

Each TrwB_*R*388_ monomer looks like an orange slice and can be divided into the NBD and AAD domains (Figure 4B). Nucleotide-binding pockets are located in the intermonomeric contact surface (these contact sites represent 25% of the total surface). Therefore, in the hexameric conformation, 50% of the surface of each subunit is in contact with neighboring subunits. The crystal structures solved in the presence of nucleotides (Gomis-Rüth et al., 2002b) show conformational movements in the active site which extend through the ICH. Then, nucleotide binding or hydrolysis can trigger conformational changes in the T4CP, thus facilitating interactions with the substrate (Gomis-Rüth et al., 2002b).

**FIGURE 5:**
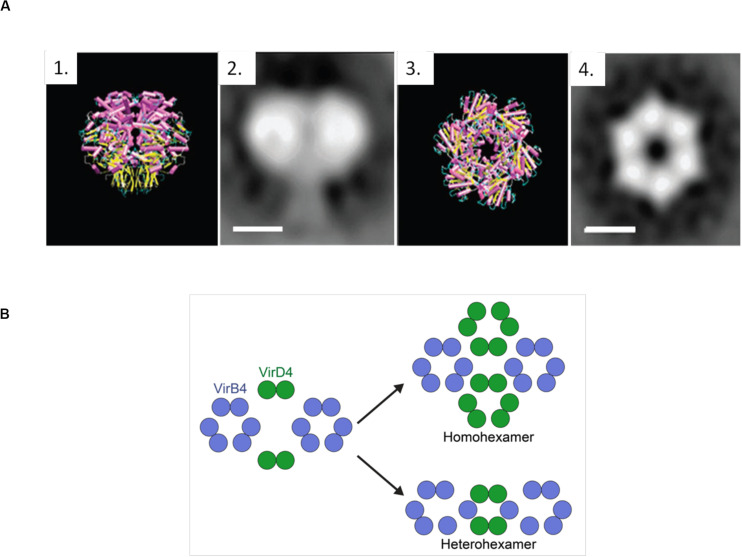
**(A)** TrwBΔN70 and TrwB_*R*388_ hexamers. 1 and 3 represent the side and top view of TrwBΔN70, respectively, solved by X-ray diffraction. 2 and 4 represent the side and top view of TrwB_*R*388_, respectively, obtained by electron microscopy. Scale bar: 50 Å. Figure adapted from Hormaeche et al. (2002). **(B)** Representation of the possible functional oligomeric states of T4CPs. It has been described that T4CPs interact with the T4SS as dimers (Redzej et al., 2017). As their functional structure is postulated to be a hexamer, they could perform their role as (i) homohexamers, composed of six T4CP subunits; or (ii) heterohexamers, composed of four T4CP subunits and two VirB4 subunits.

Negative-stain electron microscopy images (NS-EM) (Hormaeche et al., 2002) of the complete TrwB_*R*388_ reflect a strong structural similarity to TrwBΔN70, as well as a 25 Å-sized appendix that corresponds to the TMD (Figure 5A). Similarly, Redzej et al. (2017) studied TrwB_*R*388_, along with the T4SS_*R*388_ complex, by NS-EM, observing that two TrwB_*R*388_ dimers are localized between two TrwK_*R*388_ hexamers. It must be taken into account that fixed images provided by Ns-EM cannot properly represent all the steps involved in the conjugative process, where interactions between substrates and T4SSs are highly dynamic and complex. It is possible that the abovementioned NS-EM images represent only one of the steps of the transfer process. In this regard, some authors (Li et al., 2019) have claimed that the hexameric structure solved for TrwBΔN70 does not correspond to the real *in vivo* oligomeric and functional state.

#### Oligomerization

The functional structure of T4CPs is postulated to be a homohexamer that presents wide contact regions in the cytoplasmic part of the monomers. However, the results presented by Redzej et al. (2017) suggest that the *in vivo* functional state might be dynamic in the sense of possible switching from dimers to hexamers (Figure 5B). Also, from combined electron microscopy and docking calculations, it has been postulated (Peña et al., 2012; Waksman, 2019) that its active form could consist of heterohexamers with VirB4-like proteins. This interesting hypothesis requires further exploration but opens up a road for computational modeling and biophysical studies regarding the possibility of (hetero)-dimerization interfaces as crucial sites for the inhibition of conjugation. Additionally, through electron cryotomography, a technique most useful for the analysis of secretion systems (Oikonomou and Jensen, 2019), density images of T4CPs from Dot/IcmT4SS and CagT4SS (DotL_*Lp*_ and Cagβ_*Hp*_, respectively) have been obtained (Chetrit et al., 2018; Hu et al., 2019b; Li et al., 2019). The discrepancies shown by the *in vivo* oligomeric states of these T4CPs underline the need for further studies on this matter.

Nevertheless, the requirement of the TMD for oligomerization has been highlighted in different studies (Hormaeche et al., 2002; Schroder and Lanka, 2003; Mihajlovic et al., 2009). Precisely, it seems that interactions in the TMD are held by key amino acids located in the second helix of the TMD (Segura et al., 2013), specifically the GXXXXG motif which is related to the oligomerization of many membrane proteins (Teese and Langosch, 2015). In fact, the mutant protein TrwBΔN70 cannot be purified as a hexamer and only *in vitro* assays in which TrwBΔN70 interacts with G-quadruplex DNA (G4-DNA) show oligomers (Matilla et al., 2010). Other studies (Haft et al., 2007) indicate that interaction with DNA is not necessary for *in vivo* oligomerization of TraD_*F*_. According to this TraD_*F*_ oligomerization model, the T4CP enters the membrane as a monomer and then establishes contact with the TMD of another monomer. Thus, homodimers are formed by CTM-mediated interactions. Finally, accessory proteins encoded in the F-plasmid recognize N-terminal sequences and favor the creation of trimers of dimers, thus obtaining membrane-stabilized hexamers. In the absence of the TMD, the cytosolic domains of TraD_*F*_ monomers do not interact, highlighting the relevance of the TMD for oligomerization. In any case, the oligomerization mechanism of T4CPs lacking TMD, such as TraJ_*pIP*501_, is unknown. Despite some evidence for their dimerization, the hexameric form of TraJ_*pIP*501_ has not been observed so far (Abajy et al., 2007).

### Molecular Interactions

Type IV coupling proteins interact with both T4SS-proteins and the substrate to be transferred, creating a complex interaction network which is thought to be highly dynamic during the conjugative process itself, possibly owing to the presence of different signals (Christie, 2016; Redzej et al., 2017). As an example, it is a well-known fact that the cytosolic domain interacts with the substrate and/or the accessory proteins of the relaxosome. Interactions with the relaxase are indeed specific, but less tight than those with the accessory proteins of the relaxosome or molecular chaperones (Sastre et al., 1998; Hamilton et al., 2000; Llosa et al., 2003). Additionally, highly regulated interactions between the cytosolic domain of T4CPs and DNA have been described both *in vivo* and *in vitro*. These interactions are independent of the DNA sequence (Moncalián et al., 1999; Abajy et al., 2007). *In vivo* assays suggest that they depend on DNA processing and MOB protein activity (Cascales and Christie, 2004a; Chen et al., 2008). Moreover, the DNA structure seems a relevant factor for interaction specificity, as TrwBΔN70 shows higher affinity for G4-DNA than for DNA molecules lacking this particular structure (Matilla et al., 2010). On the other hand, interactions between TraG_*RP*4_ and DNA can be inhibited *in vivo* with ATP, ADP, and Mg^2+^ (Schroder and Lanka, 2003). T4CPs of T4BSS have also shown binding to DNA, although they are only implicated in effector protein transport (Schroder et al., 2002).

In addition, TMD and cytosolic domains are involved in interactions with the T4SS. The cytosolic side of T4CPs interacts with other ATPases that form the energy center (i.e., VirB4 and VirB11-like proteins) (Llosa and Alkorta, 2017) and TMD interacts with VirB10-like proteins (Segura et al., 2013). This non-specific interaction occurs between the periplasmic loops (Llosa et al., 2003; Gunton et al., 2005). Moreover, the T4CP can interact with heterologous T4SSs, creating an opportunity to form chimeric conjugation or effector-translocator systems (de Paz et al., 2005). Finally, in Gram-negative bacteria, T4CPs can interact with peptidoglycan hydrolases (Alvarez-Martinez and Christie, 2009).

### Function

There is no consensus regarding the possible roles of the T4CP during conjugation. When the crystal structure of TrwB_*R*388_ was first solved, it was proposed (Gomis-Rüth et al., 2001) that, apart from having a role in the coupling between the substrate and the T4SS, it could also function as a molecular motor. Nevertheless, since then, four additional roles have been proposed highlighting the importance of T4CPs for conjugation: (i) substrate reception to the T4SS; (ii) energy source for conjugation; (iii) signal transduction; and (iv) substrate translocation. All domains appear to be relevant; actually, the lack of any domain or the presence of mutations have important effects on conjugation frequency (De Paz et al., 2010; Cascales et al., 2013; Whitaker et al., 2015). Specifically, mutants lacking the TMD or NBD cannot accomplish conjugation, underlining the relevance of the TMD (i.e., subcellular location, regulation, interactions with the T4SS) and the ATPase activity (Moncalián et al., 1999; Kumar and Das, 2002; Gunton et al., 2005; Chen et al., 2008; Lang and Zechner, 2012). Indeed, the ATPase activity of the T4CP is essential for conjugation (Cascales et al., 2013). What is more, as mentioned before, punctual mutations on the NBD can inhibit conjugation (Tato et al., 2005). However, so far, this ATPase activity has not been described in the native protein, probably because of the highly complex regulatory network existing in the bacterial cell which can include accessory proteins, interactions with lipids, and unknown signal-mediated conformational changes. Along these lines, it has been reported (Cascales et al., 2013) that T4CPs take part in the complex signaling network happening during conjugation through their interactions with the relaxosome and the channel subunits.

Pertaining to the essential role of the T4CP in substrate transfer, it has been demonstrated (Draper et al., 2005; De Paz et al., 2010) that, in the absence of DNA transfer, the ATPase activity of the T4CP is required for the transport of the relaxase. On the other hand, TrIP studies have demonstrated that, upon the activation of conjugation, the T4CP is the first protein to interact with the ssDNA substrate prior to its delivery to VirB11 (Cascales and Christie, 2004b).

Finally, considering the structural homology of T4CPs with other DNA transfer motors, as well as their interactions with the substrate, it has been suggested (Cabezon and de la Cruz, 2006) that T4CPs could act as motors that pump the ssDNA through its ICH, taking advantage of their ATPase activity, Nonetheless, no key amino acids have been clearly identified to perform such ssDNA transport (Larrea et al., 2017).

## Inhibition of T4CPs to Control Antibiotic Resistance Dissemination

### Inhibition of Conjugation

Bacterial conjugation is the main process by which bacteria acquire (i) resistance to antibiotics; and (ii) the ability to transfer such resistance to other bacteria, thus contributing to the spread of antibiotic resistance. Therefore, a possible strategy to control the spread of antibiotic resistance is to inhibit the activity of conjugative proteins. However, inhibition of bacterial conjugation has received little attention as the search for new antibiotics has captured most of the scientific interest (Kwapong et al., 2019).

Since the T4SS has, at least, 12 different proteins, the possibilities for finding therapeutic targets (inhibitors of conjugative proteins and, hence, conjugation itself) are broad. A detailed biochemical and biophysical study of the key elements in the conjugative process is required to be able to properly assess the potential of such strategy. In the last decade, a lot of research (see below) has been conducted in the search for specific inhibitors of key conjugative proteins, without interfering with other processes present in human cells or the environment. In particular, the search for conjugation inhibitors has focused on two key proteins: (i) the VirB8-like proteins and (ii) VirB11-like proteins (one of the ATPases of the T4SS).

VirB8-like proteins are essential assembly factors that play a key role in protein-protein interactions in all T4SS. They are needed for substrate translocation from the cytoplasm to the recipient bacterium. Therefore, their specific inhibition could control the dissemination of antibiotic resistance in a targeted way (Baron, 2006). Based on structural data of TraE_*pKM*101_ (the VirB8-like protein of plasmid pKM101), the surface groove located between the α-helical and the β-sheet faces was used as docking site to identify and then develop small molecules capable of inhibiting the dimerization of TraE_*pKM*101_ and the conjugative transfer of plasmid pKM101 (Casu et al., 2016, 2017). This work proved the possibility of finding conjugation inhibitors by using structural information of T4SS proteins. In any event, it is still highly necessary to identify molecules that can inhibit most members of this VirB8-like protein family, regardless of their particular conjugative system.

Regarding VirB11-like proteins, two members of this family have been considered as drug targets in the search for specific conjugation inhibitors: Cagα and TrwD_*R*388_. Compounds that inhibit both the *in vitro* ATPase activity of Cagα (an essential protein for *Helicobacter pylori* virulence) and the transfer of the CagA effector protein have been identified (Hilleringmann et al., 2006). Additionally, based on virtual high throughput screening (HTS), a series of 8-amino imidazo(1,2-a)pyrazine derivatives that inhibit the ATPase of Cagα have been identified (Sayer et al., 2014). Unfortunately, in both studies (Hilleringmann et al., 2006; Sayer et al., 2014), there are concerns about the specificity of these compounds since they may also inhibit other bacterial or mammalian ATPases. Alternatively, an approach that does not specifically target Cagα ATPase activity has been tested (Arya et al., 2019), finding molecules that bind and stabilize Cagα, some of which inhibited Cagα ATPase activity and also dissociated Cagα hexamers.

Unsaturated and alkynoic fatty acid derivatives (Fernandez-Lopez et al., 2005; Getino et al., 2015) have also been found to effectively inhibit bacterial conjugation. Similarly, tanzawaic acids have potential as conjugation inhibitors (Getino et al., 2016). In particular, TrwD_*R*388_, the VirB11 analog of R388 plasmid, has been the target of the abovementioned fatty acids and some derivatives, such as 2-bromopalmitic acid (Ripoll-Rozada et al., 2016; García-Cazorla et al., 2018).

All these studies support the possibility of using specific inhibitors of key conjugative proteins in order to control the spread of antibiotic resistance among bacteria. Considering the well-known current magnitude of the antibiotic resistance problem, we must urgently test the widest possible repertoire of therapeutic targets in the search for specific inhibitors of bacterial conjugation. In that line of action, here we have described (i) the key role played by T4CPs; (ii) the different members of this family; and (iii) the functional and structural characteristics of those members (most relevantly, TrwB_*R*388_) on which there is information.

### Computational Approaches for the Identification and Design of T4SS Protein Inhibitors

Computational approaches applied to tackle biological problems encompass multiple techniques, such as molecular docking, virtual screening, and molecular dynamics simulations, among others (Martín-Santamaría, 2018). These techniques have proven to be very useful for the study of molecular recognition processes, as well as for the identification and design of a vast variety of compounds with biological activity (Leelananda and Lindert, 2016). Computational methods are especially helpful in the early stages of the search for novel modulators, e.g., prior to HTS, but also to understand the functioning of biological macromolecules, thus providing clues for the identification and design of novel molecules with potential as therapeutic agents.

**FIGURE 6:**
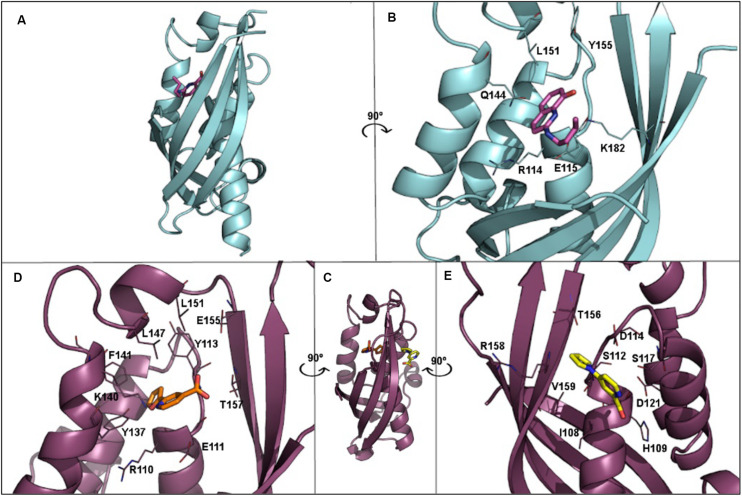
**(A)** 3D structure of VirB8 from *Brucella suis* (cyan) in complex with inhibitor B8I-1 (2-(butylamino)-8-quinolinol, magenta) (PDB ID 4AKY). **(B)** Detail of key residues at the binding site from PDB ID 4AKY. **(C)** 3D structure of TraE_*pKM*101_ from *Escherichia coli* (plum) in complex with inhibitor 239852 (orange) bound at the groove (PDB ID 5WIP) and superimposed to the 3D structure of *E. coli* TraE_*pKM*101_ (also in plum) in complex with inhibitor 105055 (yellow) bound to the α-helical region (PDB ID 5WIO). **(D)** Detail of key residues at the binding site from PDB ID 5WIP. **(E)** Detail of key residues at the binding site from PDB ID 5WIO.

T4SS proteins have been studied by computational methods in order to (i) better understand their mechanism of action; and (ii) obtain inhibitors to modulate their functions. In this respect, salicylidene acylhydrazide-type compounds were described (Smith et al., 2012) as inhibitors of *Brucella suis* VirB8 upon binding to a pocket away from the dimerization interface. The binding of these compounds prevented dimerization in both bacterial two-hybrid (BTH) assays and fluorescence resonance energy transfer-based *in vitro* assays, suggesting an allosteric mechanism of inhibition (Smith et al., 2012). The identification of the binding pocket was assisted by molecular docking of ligands B8I-1 to B8I-20 (Figure 6 and Table 2). The binding takes place in the pocket formed by residues R_114_, E_115_, Q_144_, L_151_, Y_155_, and K_182_ (Figures 6A,B). Based on these results, B8I-2 derivatives were synthesized and two of them (UM0125823 and UM0125848, Table 2) were found to be active against VirB8 dimerization. Docking prediction for these derivatives was in agreement with experimental data. Some of these compounds (B8I-1 to B8I-16 and UM-023 to UM-050) were later tested against TraE_*pKM*101_, along with other novel BI8-2 derivatives (Casu et al., 2016). The binding site (mainly, R_110_, E_111_, K_140_, and T_157_ residues; Figures 6C,D), a cavity homologous to the groove present in VirB8, was identified by means of molecular docking. The predicted binding for these compounds agreed with *in vitro* and *in vivo* experimental data: B8I-16, BAR-072, BAR-073, and UM-024 (Table 2) showed reduced TraE dimerization and pKM101 transfer.

**TABLE 2:**
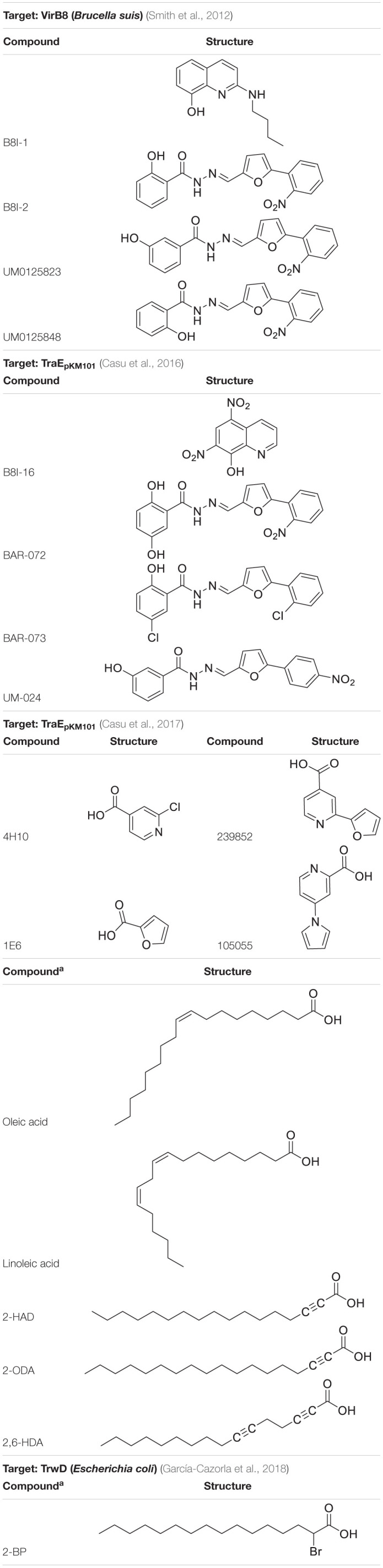
Chemical structure of ligands reported as T4SS protein inhibitors.

*^a^IUPAC names. oleic acid, (Z)-octadec-9-enoic acid; linoleic acid, (9Z,12Z)-octadeca-9,12-dienoic acid; 2-HDA, hexadec-2-ynoic acid; 2-ODA, octadec-2-ynoic acid; 2,6-HDA, hexadeca-2,6-diynoic acid; 2-BP, 2-bromo-hexadecanoic acid.*

Other VirB8-type protein inhibitors have been observed to inhibit TraE_*pKM*101_ upon fragment-based screening using a differential scanning fluorimetry assay. Possible binding sites for the fragments were identified by means of molecular docking and X-ray crystallography (Casu et al., 2017). Docking calculations showed that many fragments bind to the surface groove previously reported (Smith et al., 2012; Casu et al., 2016), but also to a novel binding site located at the α-helical region (H_109_, S_112_, D_121_, and V_159_ residues; Figure 6E) near to the dimerization interface. For two of the fragments, 4H10 and 1E6 (Table 2), X-ray structures, in complex with TraE, were obtained (PDB ID 5WII and 5WIC), showing that 1E6 binds to both binding sites while 4H10 binds near to the groove. Based on these fragments, derivatives 105055 and 239852 (Table 2) were found and docked, predicting improved affinity. The prediction was confirmed by X-ray crystallography (PDB ID 5WIO, in complex with 105055, and 5WIP, with 239852), which showed binding to the surface groove for 239852 (Figure 6D) and to the α-helical region of TraE for 105055 (Figure 6E). Further biological assays confirmed the inhibition of conjugative transfer of pKM101.

Some fatty acids, e.g., linoleic and oleic acid (Fernandez-Lopez et al., 2005), and 2-alkynoic fatty acid derivatives (Table 2), such as 2-hexadecynoic acid (2-HDA), 2-octadecynoic acid (2-ODA) and 2,6-hexadecadiynoic acid (2,6-HDA) (Getino et al., 2015), have been reported to inhibit bacterial conjugation. These fatty acids have been identified to target the VirB11-like protein TrwD_*R*388_ of *Escherichia coli* (Ripoll-Rozada et al., 2016), inhibiting TrwD function without competing with its endogenous substrates. To understand the inhibition mechanisms of these fatty acids, a homology model of TrwD was built with using *B. suis* VirB11 as a template (Ripoll-Rozada et al., 2016). Subsequent docking studies were performed, showing that the fatty acids could bind the N-terminal domain (residues 37–54) and the linker region (residues 118–125), thus affecting the plasticity of the N-terminal domain and, hence, avoiding the conformational changes needed for ATPase activity. The docking predictions also reported the same binding site for palmitic acid. Further studies (García-Cazorla et al., 2018) have shown that the fatty acids abovementioned as TrwD inhibitors, as well as a novel inhibitor, 2-bromopalmitic acid (2-BP, Table 2), can be incorporated to the bacterial membrane and affect TrwD binding upon competition with palmitic acid. Docking studies for 2-BP showed the same binding site than that for linoleic, oleic and palmitic acid, but with a different binding mode.

## Concluding Remarks

Bacterial conjugation, the major mechanism responsible for the acquisition of new genes by bacteria, is one of the main processes responsible for the dissemination of antibiotic resistance among bacteria. Conjugation is performed by T4SSs, which provide substrate processing and its transfer to the recipient bacterium through a macromolecular complex composed of at least 12 different proteins. The T4CP, one of the essential proteins of this process, is present in all conjugative systems. Despite their importance, few T4CPs have been studied in detail, being TrwB_*R*388_ the structural and functional paradigm of the family. Along with TrwB_*R*388_, in the scientific literature, there are data on the structure, oligomerization, function and interactions with other T4SS proteins for a few members of the T4CP family, which have been described here.

The worrying increase in the emergence and dissemination of antibiotic multi-resistant bacteria, together with the loss of effectiveness of current antibiotics, highlights the great urgency to find effective strategies to control antibiotic resistance spread. The search for inhibitors of key conjugative proteins, using *in vitro* HTS and computational approaches, has proved to be extremely useful to identify potential inhibitors of bacterial conjugation. This review aims to compile the progress made so far in the knowledge of T4CPs, from a structural and molecular viewpoint, as well as the work performed to identify and design conjugation inhibitors through the specific inhibition of key conjugative proteins. The combination of computational approaches with the existing body of knowledge on T4CPs will facilitate the development of conjugation inhibitors targeting T4CPs, with the ultimate goal of selectively controlling the spread of antibiotic resistance. To this purpose, further biophysical and computational studies on T4CP function, interactions between T4CPs and T4SS proteins, and dimerization/oligomerization among T4CP homologues are required.

## Author Contributions

IÁ-R, SM-S, CG, and IA: design of the work (text and figures) and literature review, writing and revision of the manuscript, and approval of the last version. EG-R, and SM-S: literature review, writing and design of figures and tables, and approval of the last version. BU-U and LA: writing and design of figures, revision of the manuscript, and approval of the last version. All authors contributed to the article and approved the submitted version.

## Conflict of Interest

The authors declare that the research was conducted in the absence of any commercial or financial relationships that could be construed as a potential conflict of interest.

## References

[B1] Abajy M. Y. Kopeć J. Schiwon K. Burzynski M. Döring M. Bohn C. (2007). A type IV-secretion-like system is required for conjugative DNA transport of broad-host-range plasmid pIP501 in gram-positive bacteria. *J. Bacteriol.* 189 2487–2496. 10.1128/jb.01491-06 17209024 PMC1899387

[B2] Águila-Arcos S. Álvarez-Rodríguez I. Garaiyurrebaso O. Garbisu C. Grohmann E. Alkorta I. (2017). Biofilm-forming clinical Staphylococcus isolates harbour horizontal transfer and antibiotic resistance genes. *Front. Microbiol.* 8:2018. 10.3389/fmicb.2017.02018 29085354 PMC5650641

[B3] Alvarez-Martinez C. E. Christie P. J. (2009). Biological diversity of prokaryotic type IV secretion systems. *Microbiol. Mol. Biol. Rev.* 73 775–808. 10.1128/mmbr.00023-09 19946141 PMC2786583

[B4] Arya T. Oudouhou F. Casu B. Bessette B. Sygusch J. Baron C. (2019). Fragment-based screening identifies inhibitors of ATPase activity and of hexamer formation of Cagα from the *Helicobacter* pylori type IV secretion system. *Sci. Rep.* 9:6474. 10.1038/s41598-019-42876-6 PMC6482174 31019200

[B5] Aussel L. Barre F. X. Aroyo M. Stasiak A. Stasiak A. Z. Sherratt D. (2002). FtsK is a DNA motor protein that activates chromosome dimer resolution by switching the catalytic state of the XerC and XerD recombinases. *Cell* 108 195–205. 10.1016/s0092-8674(02)00624-4 11832210

[B6] Baron C. (2006). VirB8: a conserved type IV secretion system assembly factor and drug target. *Biochem. Cell Biol.* 25 890–899. 10.1139/o06-148 17215876

[B7] Basu U. Lee S.-W. Deshpande A. Shen J. Sohn B.-K. Cho H. (2020). The C-terminal tail of the yeast mitochondrial transcription factor Mtf1 coordinates template strand alignment, DNA scrunching and timely transition into elongation. *Nucleic Acids Res.* 48 2604–2620. 10.1093/nar/gkaa040 31980825 PMC7049685

[B8] Bellanger X. Payot S. Leblond-Bourget N. Guédon G. (2014). Conjugative and mobilizable genomic islands in bacteria: evolution and diversity. *FEMS Microbiol. Rev.* 38 720–760. 10.1111/1574-6976.12058 24372381

[B9] Bello-López J. M. Cabrero-Martínez O. A. Ibáñez-Cervantes G. Hernández-Cortez C. Pelcastre-Rodríguez L. I. Gonzalez-Avila L. U. (2019). Horizontal gene transfer and its association with antibiotic resistance in the genus aeromonas spp. *Microorganisms* 7:363. 10.3390/microorganisms7090363 31540466 PMC6780555

[B10] Bhatty M. Gomez J. A. L. Christie P. J. (2013). The expanding bacterial type IV secretion lexicon. *Res. Microbiol.* 164 620–639. 10.1016/j.resmic.2013.03.012 23542405 PMC3816095

[B11] Cabezon E. de la Cruz F. (2006). TrwB: an F1-ATPase-like molecular motor involved in DNA transport during bacterial conjugation. *Res. Microbiol.* 157 299–305. 10.1016/j.resmic.2005.12.002 16427770

[B12] Cabezón E. Lanka E. de la Cruz F. (1994). Requirements for mobilization of plasmids RSF1010 and ColE1 by the IncW plasmid R388: trwB and RP4 traG are interchangeable. *J. Bacteriol.* 176 4455–4458. 10.1128/jb.176.14.4455-4458.1994 8021231 PMC205661

[B13] Cascales E. Atmakuri K. Sarkar M. K. Christie P. J. (2013). DNA substrate-induced activation of the agrobacterium VirB/VirD4 Type IV secretion system. *J. Bacteriol.* 195 2691–2704. 10.1128/jb.00114-13 23564169 PMC3676061

[B14] Cascales E. Christie P. J. (2004a). *Agrobacterium* VirB10, an ATP energy sensor required for type IV secretion. *Proc. Natl. Acad. Sci. U.S.A.* 101 17228–17233. 10.1073/pnas.0405843101 15569944 PMC535377

[B15] Cascales E. Christie P. J. (2004b). Definition of a bacterial type IV secretion pathway for a DNA substrate. *Science* 304 1170–1173. 10.1126/science.1095211 15155952 PMC3882297

[B16] Cassini A. Högberg L. D. Plachouras D. Quattrocchi A. Hoxha A. Simonsen G. S. (2019). Attributable deaths and disability-adjusted life-years caused by infections with antibiotic-resistant bacteria in the EU and the European Economic Area in 2015: a population-level modelling analysis. *Lancet Infect. Dis.* 19 56–66. 30409683 10.1016/S1473-3099(18)30605-4 PMC6300481

[B17] Casu B. Arya T. Bessette B. Baron C. (2017). Fragment-based screening identifies novel targets for inhibitors of conjugative transfer of antimicrobial resistance by plasmid pKM101. *Sci. Rep.* 7:14907. 10.1038/s41598-017-14953-1 PMC5668240 29097752

[B18] Casu B. Smart J. Hancock M. A. Smith M. Sygusch J. Baron C. (2016). Structural Analysis and Inhibition of TraE from the pKM101 Type IV Secretion System. *J. Biol. Chem.* 291 23817–23829. 10.1074/jbc.m116.753327 27634044 PMC5095433

[B19] Chan M. (2015). WHO Library Cataloguing-in-Publication Data Global Action Plan on Antimicrobial Resistance, ed. World Health Organization. 10.1074/jbc.m116.753327 27634044 PMC5095433

[B20] Chen Y. Zhang X. Manias D. Yeo H.-J. Dunny G. M. Christie P. J. (2008). *Enterococcus faecalis* PcfC, a spatially localized substrate receptor for type iv secretion of the pcf10 transfer intermediate. *J. Bacteriol.* 190 3632–3645. 10.1128/jb.01999-07 18326569 PMC2394995

[B21] Chetrit D. Hu B. Christie P. J. Roy C. R. Liu J. (2018). A unique cytoplasmic ATPase complex defines the *Legionella pneumophila* type IV secretion channel. *Nat. Microbiol.* 3 678–686. 10.1038/s41564-018-0165-z 29784975 PMC5970066

[B22] Christie P. J. (2004). Type IV secretion: the agrobacterium VirB/D4 and related conjugation systems. *Biochim. Biophys. Acta Mol. Cell Res.* 1694 219–234. 10.1016/j.bbamcr.2004.02.013 15546668 PMC4845649

[B23] Christie P. J. (2016). The mosaic type IV secretion systems. *EcoSal Plus* 7:10.1128/ecosalplus.ESP-0020-2015. 10.1128/ecosalplus.ESP-0020-2015 27735785 PMC5119655

[B24] Christie P. J. Atmakuri K. Krishnamoorthy V. Jakubowski S. Cascales E. (2005). Biogenesis, architecture, and function of bacterial type IV secretion systems. *Annu. Rev. Microbiol.* 59 451–485. 10.1146/annurev.micro.58.030603.123630 16153176 PMC3872966

[B25] Christie P. J. Gomez Valero L. Buchrieser C. (2017). Biological diversity and evolution of Type IV secretion systems. *Curr. Top. Microbiol. Immunol.* 413 1–30. 10.1007/978-3-319-75241-9_1 29536353 PMC5912172

[B26] De Paz H. D. Larrea D. Zunzunegui S. Dehio C. De La Cruz F. Llosa M. (2010). Functional dissection of the conjugative coupling protein TrwB. *J. Bacteriol.* 192 2655–2669. 10.1128/jb.01692-09 20363945 PMC2876477

[B27] de Paz H. D. Sangari F. J. Bolland S. García-Lobo J. M. Dehio C. de la Cruz F. (2005). Functional interactions between type IV secretion systems involved in DNA transfer and virulence. *Microbiology* 151 3505–3516. 10.1099/mic.0.28410-0 16272374

[B28] Draper O. César C. E. Machón C. de la Cruz F. Llosa M. (2005). Site-specific recombinase and integrase activities of a conjugative relaxase in recipient cells. *Proc. Natl. Acad. Sci. U.S.A.* 102 16385–16390. 10.1073/pnas.0506081102 16260740 PMC1283433

[B29] Fernandez-Lopez R. Machón C. Longshaw C. M. Martin S. Molin S. Zechner E. L. (2005). Unsaturated fatty acids are inhibitors of bacterial conjugation. *Microbiology* 151 3517–3526. 16272375 10.1099/mic.0.28216-0

[B30] Frost L. S. Leplae R. Summers A. O. Toussaint A. (2005). Mobile genetic elements: the agents of open source evolution. *Nat. Rev. Microbiol.* 3 722–732. 10.1038/nrmicro1235 16138100

[B31] Garbisu C. Garaiyurrebaso O. Lanzén A. Álvarez-Rodríguez I. Arana L. Blanco F. (2018). Mobile genetic elements and antibiotic resistance in mine soil amended with organic wastes. *Sci. Total Environ.* 621 725–733. 10.1016/j.scitotenv.2017.11.221 29207350

[B32] García-Cazorla Y. Getino M. Sanabria-Ríos D. J. Carballeira N. M. de la Cruz F. Arechaga I. (2018). Conjugation inhibitors compete with palmitic acid for binding to the conjugative traffic ATPase TrwD, providing a mechanism to inhibit bacterial conjugation. *J. Biol. Chem.* 293 16923–16930. 10.1074/jbc.ra118.004716 30201608 PMC6204903

[B33] Garcillán-Barcia M. P. Francia M. V. de La Cruz F. (2009). The diversity of conjugative relaxases and its application in plasmid classification. *FEMS Microbiol. Rev.* 33 657–687. 10.1111/j.1574-6976.2009.00168.x 19396961

[B34] Geourjon C. Orelle C. Steinfels E. Blanchet C. Deléage G. Di Pietro A. (2001). A common mechanism for ATP hydrolysis in ABC transporter and helicase superfamilies. *Trends Biochem. Sci.* 26 539–544. 10.1016/s0968-0004(01)01907-7 11551790

[B35] Getino M. Fernández-López R. Palencia-Gándara C. Campos-Gómez J. Sánchez-López J. M. Martínez M. (2016). Tanzawaic acids, a chemically novel set of bacterial conjugation inhibitors. *PLoS One* 11:148098. 10.1371/journal.pone.0148098 26812051 PMC4727781

[B36] Getino M. Sanabria-Ríos D. J. Fernández-López R. Campos-Gómez J. Sánchez-López J. M. Fernández A. (2015). Synthetic fatty acids prevent plasmid-mediated horizontal gene transfer. *mBio* 6:e0032-15. 10.1128/mBio.01032-15 PMC4556808 26330514

[B37] Gillings M. Boucher Y. Labbate M. Holmes A. Krishnan S. Holley M. (2008). The evolution of class 1 integrons and the rise of antibiotic resistance. *J. Bacteriol.* 190 5095–5100. 10.1128/jb.00152-08 18487337 PMC2447024

[B38] Gomis-Ruth F. Sola M. Cruz F. Coll M. (2005). Coupling factors in macromolecular Type-IV secretion machineries. *Curr. Pharm. Des.* 10 1551–1565. 10.2174/1381612043384817 15134575

[B39] Gomis-Rüth F. X. Coll M. (2006). Cut and move: protein machinery for DNA processing in bacterial conjugation. *Curr. Opin. Struct. Biol.* 16 744–752. 10.1016/j.sbi.2006.10.004 17079132

[B40] Gomis-Rüth F. X. de la Cruz F. Coll M. (2002a). Structure and role of coupling proteins in conjugal DNA transfer. *Res. Microbiol.* 153 199–204. 10.1016/s0923-2508(02)01313-x 12066890

[B41] Gomis-Rüth F. X. Moncalián G. De La Cruz F. Coll M. (2002b). Conjugative plasmid protein TrwB, an integral membrane type IV secretion system coupling protein. Detailed structural features and mapping of the active site cleft. *J. Biol. Chem.* 277 7556–7566. 10.1074/jbc.m110462200 11748238

[B42] Gomis-Rüth F. X. Moncalián G. Pérez-Luque R. González A. Cabezón E. de la Cruz F. (2001). The bacterial conjugation protein TrwB resembles ring helicases and F1-ATPase. *Nature* 409 637–641. 10.1038/35054586 11214325

[B43] González-Rivera C. Khara P. Awad D. Patel R. Li Y. G. Bogisch M. (2019). Two pKM101-encoded proteins, the pilus-tip protein TraC and Pep, assemble on the *Escherichia coli* cell surface as adhesins required for efficient conjugative DNA transfer. *Mol. Microbiol.* 111 96–117. 10.1111/mmi.14141 30264928 PMC6351158

[B44] Gordon J. E. Costa T. R. D. Patel R. S. Gonzalez-Rivera C. Sarkar M. K. Orlova E. V. (2017). Use of chimeric type IV secretion systems to define contributions of outer membrane subassemblies for contact-dependent translocation. *Mol. Microbiol.* 105 273–293. 10.1111/mmi.13700 28452085 PMC5518639

[B45] Grohmann E. Christie P. J. Waksman G. Backert S. (2018). Type IV secretion in Gram-negative and Gram-positive bacteria. *Mol. Microbiol.* 107 455–471. 10.1111/mmi.13896 29235173 PMC5796862

[B46] Grohmann E. Goessweiner-Mohr N. Brantl S. (2016). DNA-binding proteins regulating pIP501 transfer and replication. *Front. Mol. Biosci.* 3:42. 10.3389/fmolb.2016.00042 PMC4981023 27563645

[B47] Grohmann E. Keller W. Muth G. (2017). Mechanisms of conjugative transfer and Type IV secretion-mediated effector transport in gram-positive bacteria. *Curr. Top. Microbiol. Immunol.* 413 115–141. 10.1007/978-3-319-75241-9_5 29536357

[B48] Guédon G. Libante V. Coluzzi C. Payot S. Leblond-Bourget N. (2017). The obscure world of integrative and mobilizable elements, highly widespread elements that pirate bacterial conjugative systems. *Genes* 8:337. 10.3390/genes8110337 29165361 PMC5704250

[B49] Guglielmini J. de la Cruz F. Rocha E. P. C. (2013). Evolution of conjugation and type IV secretion systems. *Mol. Biol. Evol.* 30 315–331. 10.1093/molbev/mss221 22977114 PMC3548315

[B50] Gunton J. E. Gilmour M. W. Alonso G. Taylor D. E. (2005). Subcellular localization and functional domains of the coupling protein, TraG, from IncHI1 plasmid R27. *Microbiology* 151 3549–3561. 10.1099/mic.0.28255-0 16272378

[B51] Gunton J. E. Gilmour M. W. Baptista K. P. Lawley T. D. Taylor D. E. (2007). Interaction between the co-inherited TraG coupling protein and the TraJ membrane-associated protein of the H-plasmid conjugative DNA transfer system resembles chromosomal DNA translocases. *Microbiology* 153 428–441. 10.1099/mic.0.2006/001297-0 17259614

[B52] Guzmán-Herrador D. L. Steiner S. Alperi A. González-Prieto C. Roy C. R. Llosa M. (2017). DNA delivery and genomic integration into mammalian target cells through Type IV A and B secretion systems of human pathogens. *Front. Microbiol.* 8:1503. 10.3389/fmicb.2017.01503 PMC5572225 28878740

[B53] Haft R. J. F. Gachelet E. G. Nguyen T. Toussaint L. Chivian D. Traxler B. (2007). In vivo oligomerization of the F conjugative coupling protein TraD. *J. Bacteriol.* 189 6626–6634. 10.1128/jb.00513-07 17631633 PMC2045173

[B54] Hamilton C. M. Lee H. Li P.-L. Cook D. M. Piper K. R. von Bodman S. B. (2000). TraG from RP4 and TraG and VirD4 from Ti Plasmids confer relaxosome specificity to the conjugal transfer system of pTiC58. *J. Bacteriol.* 182 1541–1548. 10.1128/jb.182.6.1541-1548.2000 10692358 PMC94450

[B55] Hanson P. I. Whiteheart S. W. (2005). AAA+ proteins: have engine, will work. *Nat. Rev. Mol. Cell Biol.* 6 519–529. 10.1038/nrm1684 16072036

[B56] Harrison M. L. Desaulniers M. A. Noyce R. S. Evans D. H. (2016). The acidic C-terminus of vaccinia virus I3 single-strand binding protein promotes proper assembly of DNA-protein complexes. *Virology* 489 212–222. 10.1016/j.virol.2015.12.020 26773382

[B57] Hilleringmann M. Pansegrau W. Doyle M. Kaufman S. MacKichan M. L. Gianfaldoni C. (2006). Inhibitors of *Helicobacter* pylori ATPase Cagα block CagA transport and cag virulence. *Microbiology* 152 2919–2930. 10.1099/mic.0.28984-0 17005973

[B58] Högberg L. D. Heddini A. Cars O. (2010). The global need for effective antibiotics: challenges and recent advances. *Trends Pharmacol. Sci.* 31 509–515. 10.1016/j.tips.2010.08.002 20843562

[B59] Hormaeche I. Alkorta I. Moro F. Valpuesta J. M. Goni F. M. De La Cruz F. (2002). Purification and properties of TrwB, a hexameric, ATP-binding integral membrane protein essential for R388 plasmid conjugation. *J. Biol. Chem.* 277 46456–46462. 10.1074/jbc.m207250200 12244053

[B60] Hormaeche I. Iloro I. Arrondo J. L. R. Goñi F. M. De La Cruz F. Alkorta I. (2004). Role of the transmembrane domain in the stability of TrwB, an integral protein involved in bacterial conjugation. *J. Biol. Chem.* 279 10955–10961. 10.1074/jbc.m310422200 14699106

[B61] Hormaeche I. Segura R. L. Vecino A. J. Goñi F. M. de la Cruz F. Alkorta I. (2006). The transmembrane domain provides nucleotide binding specificity to the bacterial conjugation protein TrwB. *FEBS Lett.* 580 3075–3082. 10.1016/j.febslet.2006.04.059 16678163

[B62] Hu B. Khara P. Christie P. J. (2019a). Structural bases for F plasmid conjugation and F pilus biogenesis in *Escherichia coli*. *Proct. Nat. Acad. Sci. U.S.A.* 116 14222–14227. 10.1073/pnas.1904428116 31239340 PMC6628675

[B63] Hu B. Khara P. Song L. Lin A. S. Frick-Cheng A. E. Harvey M. L. (2019b). In situ molecular architecture of the *Helicobacter pylori* cag type IV secretion system. *mBio* 10:e00849-19. 10.1128/mBio.00849-19 PMC6520456 31088930

[B64] Johnsborg O. Eldholm V. Håvarstein L. S. (2007). Natural genetic transformation: prevalence, mechanisms and function. *Res. Microbiol.* 158 767–778. 10.1016/j.resmic.2007.09.004 17997281

[B65] Johnson C. M. Grossman A. D. (2015). Integrative and conjugative elements (ICEs): what they do and how they work. *Annu. Rev. Genet.* 49 577–601. 10.1146/annurev-genet-112414-055018 26473380 PMC5180612

[B66] Kittell B. L. Helinski D. R. (1993). “Plasmid incompatibility and replication control,” in *Bacterial Conjugation*, ed. Clewell D. B. (Boston, MA: Springer), 223–242. 10.1007/978-1-4757-9357-4_8

[B67] Kumar R. B. Das A. (2002). Polar location and functional domains of the *Agrobacterium tumefaciens* DNA transfer protein VirD4. *Mol. Microbiol.* 43 1523–1532. 10.1046/j.1365-2958.2002.02829.x 11952902

[B68] Kwak M.-J. Kim J. D. Kim H. Kim C. Bowman J. W. Kim S. (2017). Architecture of the type IV coupling protein complex of *Legionella pneumophila*. *Nat. Microbiol.* 2:17114. 10.1038/nmicrobiol.2017.114 PMC6497169 28714967

[B69] Kwapong A. A. Stapleton P. Gibbons S. (2019). Inhibiting plasmid mobility: the effect of isothiocyanates on bacterial conjugation. *Int. J. Antimicrob. Agents* 53 629–636. 10.1016/j.ijantimicag.2019.01.011 30685311

[B70] Lang S. Zechner E. L. (2012). General requirements for protein secretion by the F-like conjugation system R1. *Plasmid* 67 128–138. 10.1016/j.plasmid.2011.12.014 22248924 PMC3338209

[B71] Larrea D. de Paz H. D. Matilla I. Guzmán-Herrador D. L. Lasso G. de la Cruz F. (2017). Substrate translocation involves specific lysine residues of the central channel of the conjugative coupling protein TrwB. *Mol. Genet. Genomics* 292 1037–1049. 10.1007/s00438-017-1331-3 28597316

[B72] Lawley T. Klimke W. Gubbins M. Frost L. (2003). F factor conjugation is a true type IV secretion system. *FEMS Microbiol. Lett.* 224 1–15. 10.1016/s0378-1097(03)00430-0 12855161

[B73] Lederberg J. Tatum E. L. (1946). Gene recombination in *Escherichia coli*. *Nature* 158 558. 10.1038/158558a0 21001945

[B74] Lee K. B. Thomas J. O. (2000). The effect of the acidic tail on the DNA-binding properties of the HMG1,2 class of proteins: Insights from tail switching and tail removal. *J. Mol. Biol.* 304 135–149. 10.1006/jmbi.2000.4206 11080451

[B75] Leelananda S. P. Lindert S. (2016). Computational methods in drug discovery. *Beilstein J. Org. Chem.* 12 2694–2718. 28144341 10.3762/bjoc.12.267 PMC5238551

[B76] Li Y. G. Christie P. J. (2018). “The Agrobacterium VirB/VirD4 T4SS: mechanism and architecture defined through in vivo mutagenesis and chimeric systems,” in *Agrobacterium Biology. Current Topics in Microbiology and Immunology*, Vol. 418 ed. Gelvin S. (Cham: Springer), 233–260. 10.1007/82_2018_94 PMC7011205 29808338

[B77] Li Y. G. Hu B. Christie P. J. (2019). Biological and structural diversity of type IV secretion systems. *Microbiol. Spectr.* 7:10.1128/microbiolsec.SIB-0012-2018. 10.1128/microbiolspec.psib-0012-2018 PMC6452883 30953428

[B78] Llosa M. Alkorta I. (2017). Coupling proteins in type IV secretion. *Curr. Top. Microbiol. Immunol.* 413 143–168. 10.1007/978-3-319-75241-9_6 29536358

[B79] Llosa M. Gomis-Rüth F. X. Coll M. de la Cruz Fd F. (2002). Bacterial conjugation: a two-step mechanism for DNA transport. *Mol. Microbiol.* 45 1–8. 10.1046/j.1365-2958.2002.03014.x 12100543

[B80] Llosa M. Zunzunegui S. de la Cruz F. (2003). Conjugative coupling proteins interact with cognate and heterologous VirB10-like proteins while exhibiting specificity for cognate relaxosomes. *Proc. Natl. Acad. Sci. U.S.A.* 100 10465–10470. 10.1073/pnas.1830264100 12925737 PMC193584

[B81] Low H. H. Gubellini F. Rivera-Calzada A. Braun N. Connery S. Dujeancourt A. (2014). Structure of a type IV secretion system. *Nature* 508, 550–553. 10.1038/nature13081 24670658 PMC3998870

[B82] Lu J. Frost L. S. (2005). Mutations in the C-terminal region of TraM provide evidence for in vivo TraM-TraD interactions during F-plasmid conjugation. *J. Bacteriol.* 187 4767–4773. 10.1128/jb.187.14.4767-4773.2005 15995191 PMC1169504

[B83] Lu J. Wong J. J. W. Edwards R. A. Manchak J. Frost L. S. Glover J. N. M. (2008). Structural basis of specific TraD-TraM recognition during F plasmid-mediated bacterial conjugation. *Mol. Microbiol.* 70 89–99. 10.1111/j.1365-2958.2008.06391.x 18717787

[B84] Martín-Santamaría S. (2018). *Computational Tools for Chemical Biology.* London: Royal Society of Chemistry.

[B85] Matilla I. Alfonso C. Rivas G. Bolt E. L. de la Cruz F. Cabezon E. (2010). The Conjugative DNA translocase TrwB is a structure-specific DNA-binding protein. *J. Biol. Chem.* 285 17537–17544. 10.1074/jbc.m109.084137 20375020 PMC2878518

[B86] Meir A. Chetrit D. Liu L. Roy C. R. Waksman G. (2018). Legionella DotM structure reveals a role in effector recruiting to the Type 4B secretion system. *Nat. Commun.* 9:507. 10.1038/s41467-017-02578-x PMC5802825 29410427

[B87] Middleton R. Sjölander K. Krishnamurthy N. Foley J. Zambryski P. (2005). Predicted hexameric structure of the Agrobacterium VirB4 C terminus suggests VirB4 acts as a docking site during type IV secretion. *Proc. Natl. Acad. Sci. U.S.A.* 102 1685–1690. 10.1073/pnas.0409399102 15668378 PMC547840

[B88] Mihajlovic S. Lang S. Sut M. V. Strohmaier H. Gruber C. J. Koraimann G. (2009). Plasmid R1 conjugative DNA processing is regulated at the coupling protein interface. *J. Bacteriol.* 191 6877–6887. 10.1128/jb.00918-09 19767437 PMC2772491

[B89] Moncalián G. Cabezón E. Alkorta I. Valle M. Moro F. Valpuesta J. M. (1999). Characterization of ATP and DNA binding activities of TrwB, the coupling protein essential in plasmid R388 conjugation. *J. Biol. Chem.* 274 36117–36124. 10.1074/jbc.274.51.36117 10593894

[B90] Oikonomou C. M. Jensen G. J. (2019). “Electron cryotomography of bacterial secretion systems,” in *Protein Secretion in Bacteria*, eds Sandkvist M. Cascales E. Christie P. (American Society for Microbiology), 1–12. 10.1128/microbiolspec.psib-0019-2018 PMC6452891 30953431

[B91] Parsons J. A. Bannam T. L. Devenish R. J. Rood J. I. (2007). TcpA, an FtsK/SpoIIIE homolog, is essential for transfer of the conjugative plasmid pCW3 in *Clostridium perfringens*. *J. Bacteriol.* 189 7782–7790. 17720795 10.1128/JB.00783-07 PMC2168741

[B92] Peña A. Matilla I. Martín-Benito J. Valpuesta J. M. Carrascosa J. L. De La Cruz F. (2012). The hexameric structure of a conjugative VirB4 protein ATPase provides new insights for a functional and phylogenetic relationship with DNA translocases. *J. Biol. Chem.* 287 39925–39932. 23035111 10.1074/jbc.M112.413849 PMC3501061

[B93] Redzej A. Ukleja M. Connery S. Trokter M. Felisberto-Rodrigues C. Cryar A. (2017). Structure of a VirD4 coupling protein bound to a VirB type IV secretion machinery. *EMBO J.* 36 3080–3095. 28923826 10.15252/embj.201796629 PMC5916273

[B94] Ripoll-Rozada J. García-Cazorla Y. Getino M. Machón C. Sanabria-Ríos D. de la Cruz F. (2016). Type IV traffic ATPase TrwD as molecular target to inhibit bacterial conjugation. *Mol. Microbiol.* 100 912–921. 26915347 10.1111/mmi.13359 PMC4908816

[B95] Ripoll-Rozada J. Zunzunegui S. de la Cruz F. Arechaga I. Cabezón E. (2013). Functional interactions of VirB11 traffic ATPases with VirB4 and VirD4 molecular motors in type IV secretion systems. *J. Bacteriol.* 195 4195–4201. 23852869 10.1128/JB.00437-13 PMC3754731

[B96] Sastre I. Cabezón E. de la Cruz F. (1998). The carboxyl terminus of protein TraD adds specificity and efficiency to F-plasmid conjugative transfer. *J. Bacteriol.* 180 6039–6042. 9811665 10.1128/jb.180.22.6039-6042.1998 PMC107681

[B97] Sayer J. R. Walldén K. Pesnot T. Campbell F. Gane P. J. Simone M. (2014). 2- and 3-substituted imidazo[1,2-a]pyrazines as inhibitors of bacterial type IV secretion. *Bioorgan. Med. Chem.* 22 6459–6470. 10.1016/j.bmc.2014.09.036 PMC4339681 25438770

[B98] Schroder G. Krause S. Zechner E. L. Traxler B. Yeo H.-J. Lurz R. (2002). TraG-like proteins of DNA transfer systems and of the *Helicobacter* pylori Type IV secretion system: inner membrane gate for exported substrates? *J. Bacteriol.* 184 2767–2779. 11976307 10.1128/JB.184.10.2767-2779.2002 PMC135038

[B99] Schroder G. Lanka E. (2003). TraG-Like proteins of type IV secretion systems: functional dissection of the multiple activities of TraG (RP4) and TrwB (R388). *J. Bacteriol.* 185 4371–4381. 12867445 10.1128/JB.185.15.4371-4381.2003 PMC165781

[B100] Schröder G. Lanka E. (2005). The mating pair formation system of conjugative plasmids-A versatile secretion machinery for transfer of proteins and DNA. *Plasmid* 54 1–25. 15907535 10.1016/j.plasmid.2005.02.001

[B101] Segura R. L. Águila-Arcos S. Ugarte-Uribe B. Vecino A. J. De La Cruz F. Goñi F. M. (2013). The transmembrane domain of the T4SS coupling protein TrwB and its role in protein-protein interactions. *Biochim. Biophys. Acta Biomembr.* 1828 2015–2025. 10.1016/j.bbamem.2013.05.022 23735543

[B102] Segura R. L. Águila-Arcos S. Ugarte-Uribe B. Vecino A. J. de la Cruz F. Goñi F. M. (2014). Subcellular location of the coupling protein TrwB and the role of its transmembrane domain. *Biochim. Biophys. Acta Biomembr.* 1838 223–230. 10.1016/j.bbamem.2013.08.016 24016550

[B103] Shaffer C. L. Good J. A. D. Kumar S. Krishnan K. S. Gaddy J. A. Loh J. T. (2016). Peptidomimetic small molecules disrupt type IV secretion system activity in diverse bacterial pathogens. *mBio* 7:e00221-16. 10.1128/mBio.00221-16 PMC4850256 27118587

[B104] Siguier P. Gourbeyre E. Chandler M. (2014). Bacterial insertion sequences: their genomic impact and diversity. *FEMS Microbiol. Rev.* 38 865–891. 24499397 10.1111/1574-6976.12067 PMC7190074

[B105] Smillie C. Garcillán-Barcia M. P. Francia M. V. Rocha E. P. C. de la Cruz F. (2010). Mobility of plasmids. *Microbiol. Mol. Biol. Rev.* 74 434–452. 20805406 10.1128/MMBR.00020-10 PMC2937521

[B106] Smith M. A. Coinon M. Paschos A. Jolicoeur B. Lavallée P. Sygusch J. (2012). Identification of the binding site of Brucella VirB8 interaction inhibitors. *Chem. Biol.* 19 1041–1048. 22921071 10.1016/j.chembiol.2012.07.007

[B107] Steen J. A. Bannam T. L. Teng W. L. Devenish R. J. Rood J. I. (2009). The putative coupling protein TcpA interacts with other pCW3-encoded proteins to form an essential part of the conjugation complex. *J. Bacteriol.* 191 2926–2933. 19251842 10.1128/JB.00032-09 PMC2681803

[B108] Sutherland M. C. Nguyen T. L. Tseng V. Vogel J. P. (2012). The Legionella IcmSW complex directly interacts with DotL to mediate translocation of adaptor-dependent substrates. *PLoS Pathog.* 8:e1002910. 10.1371/journal.ppat.1002910 PMC3441705 23028312

[B109] Tacconelli E. Carrara E. Savoldi A. Harbarth S. Mendelson M. Monnet D. L. (2018). Discovery, research and development of new antibiotics: the who priority list of antibiotic-resistant bacteria and tuberculosis. *Lancet Infect. Dis.* 18 318–327. 29276051 10.1016/S1473-3099(17)30753-3

[B110] Tato I. Matilla I. Arechaga I. Zunzunegui S. de la Cruz F. Cabezon E. (2007). The ATPase activity of the DNA transporter TrwB is modulated by protein TrwA: implications for a common assembly mechanism of DNA translocating motors. *J. Biol. Chem.* 282 25569–25576. 17599913 10.1074/jbc.M703464200

[B111] Tato I. Zunzunegui S. de la Cruz F. Cabezon E. (2005). TrwB, the coupling protein involved in DNA transport during bacterial conjugation, is a DNA-dependent ATPase. *Proc. Natl. Acad. Sci. U.S.A.* 102 8156–8161. 15919815 10.1073/pnas.0503402102 PMC1149453

[B112] Teese M. G. Langosch D. (2015). Role of GxxxG motifs in transmembrane domain interactions. *Biochemistry* 54 5125–5135. 26244771 10.1021/acs.biochem.5b00495

[B113] Thomas C. M. Nielsen K. M. (2005). Mechanisms of, and barriers to, horizontal gene transfer between bacteria. *Nat. Rev. Microbiol.* 3 711–721. 16138099 10.1038/nrmicro1234

[B114] Thomas C. M. Thomson N. R. Cerdeño-Tárraga A. M. Brown C. J. Top E. M. Frost L. S. (2017). Annotation of plasmid genes. *Plasmid* 91 61–67. 28365184 10.1016/j.plasmid.2017.03.006

[B115] Urra J. Alkorta I. Garbisu C. (2019a). Potential benefits and risks for soil health derived from the use of organic amendments in agriculture. *Agronomy* 9:542 10.3390/agronomy9090542

[B116] Urra J. Alkorta I. Lanzén A. Mijangos I. Garbisu C. (2019b). The application of fresh and composted horse and chicken manure affects soil quality, microbial composition and antibiotic resistance. *Appl. Soil Ecol.* 135 73–84.

[B117] Urra J. Alkorta I. Mijangos I. Epelde L. Garbisu C. (2019c). Application of sewage sludge to agricultural soil increases the abundance of antibiotic resistance genes without altering the composition of prokaryotic communities. *Sci. Total Environ.* 647 1410–1420. 30180347 10.1016/j.scitotenv.2018.08.092

[B118] Vecino A. J. de la Arada I. Segura R. L. Goñi F. M. de la Cruz F. Arrondo J. L. R. (2011). Membrane insertion stabilizes the structure of TrwB, the R388 conjugative plasmid coupling protein. *Biochim. Biophys. Acta* 1808 1032–1039. 21211515 10.1016/j.bbamem.2010.12.025

[B119] Voth D. E. Broederdorf L. J. Graham J. G. (2012). Bacterial Type IV secretion systems: versatile virulence machines. *Future Microbiol.* 7 241–257. 22324993 10.2217/fmb.11.150 PMC3563059

[B120] Waksman G. (2019). From conjugation to T4S systems in Gram-negative bacteria: a mechanistic biology perspective. *EMBO Rep.* 20:e47012. 10.15252/embr.201847012 PMC6362355 30602585

[B121] Walker J. E. Saraste M. Runswick M. J. Gay N. J. (1982). Distantly related sequences in the alpha- and beta-subunits of ATP synthase, myosin, kinases and other ATP-requiring enzymes and a common nucleotide binding fold. *EMBO J.* 1 945–951. 6329717 10.1002/j.1460-2075.1982.tb01276.x PMC553140

[B122] Walldén K. Williams R. Yan J. Lian P. W. Wang L. Thalassinos K. (2012). Structure of the VirB4 ATPase, alone and bound to the core complex of a type IV secretion system. *Proc. Natl. Acad. Sci. U.S.A.* 109 11348–11353. 22745169 10.1073/pnas.1201428109 PMC3396474

[B123] Wang Q. Zeng M. Wang W. Tang J. (2007). The HMGB1 acidic tail regulates HMGB1 DNA binding specificity by a unique mechanism. *Biochem. Biophys. Res. Commun.* 360 14–19. 17585880 10.1016/j.bbrc.2007.05.130

[B124] Whitaker N. Berry T. M. Rosenthal N. Gordon J. E. Gonzalez-Rivera C. Sheehan K. B. (2016). Chimeric coupling proteins mediate transfer of heterologous type IV effectors through the *Escherichia coli* pKM101-encoded conjugation machine. *J. Bacteriol.* 198 2701–2718. 27432829 10.1128/JB.00378-16 PMC5019051

[B125] Whitaker N. Chen Y. Jakubowski S. J. Sarkar M. K. Li F. Christie P. J. (2015). The All-alpha domains of coupling proteins from the *Agrobacterium tumefaciens* VirB/VirD4 and *Enterococcus faecalis* pCF10-encoded Type IV secretion systems confer specificity to binding of cognate DNA substrates. *J. Bacteriol.* 197 2335–2349. 25939830 10.1128/JB.00189-15 PMC4524192

[B126] World Health Organization (2015). *Global Action Plan On Antimicrobial Resistance.* Geneva: World Health Organization. 10.7196/samj.9644 26242647

[B127] World Health Organization (2019a). *Thirteenth General Programme Of Work 2019-2023.* Geneva: World Health Organization.

[B128] World Health Organization (2019b). *2019 Antibacterial Agents In Clinical Development: An Analysis Of The Antibacterial Clinical Development Pipeline.* Geneva: World Health Organization.

[B129] Zechner E. L. Lang S. Schildbach J. F. (2012). Assembly and mechanisms of bacterial type IV secretion machines. *Philos. Trans. R. Soc. B Biol. Sci.* 367 1073–1087. 10.1098/rstb.2011.0207 PMC3297438 22411979

